# Transcriptome analysis of nitrate assimilation in *Aspergillus nidulans* reveals connections to nitric oxide metabolism

**DOI:** 10.1111/j.1365-2958.2010.07363.x

**Published:** 2010-09-27

**Authors:** Thorsten Schinko, Harald Berger, Wanseon Lee, Andreas Gallmetzer, Katharina Pirker, Robert Pachlinger, Ingrid Buchner, Thomas Reichenauer, Ulrich Güldener, Joseph Strauss

**Affiliations:** 1 Fungal Genetics and Genomics Unit, Department of Applied Genetics and Cell Biology, Austrian Institute of Technology and BOKU University ViennaMuthgasse 18, 1190 Vienna, Austria; 2 Institute of Bioinformatics and Systems Biology, Helmholtz Zentrum München85764 Neuherberg, Germany; 3 Austrian Institute of TechnologyA-2444 Seibersdorf, Austria

## Abstract

Nitrate is a dominant form of inorganic nitrogen (N) in soils and can be efficiently assimilated by bacteria, fungi and plants. We studied here the transcriptome of the short-term nitrate response using assimilating and non-assimilating strains of the model ascomycete *Aspergillus nidulans*. Among the 72 genes positively responding to nitrate, only 18 genes carry binding sites for the pathway-specific activator NirA. Forty-five genes were repressed by nitrate metabolism. Because *nirA*^-^ strains are N-starved at nitrate induction conditions, we also compared the nitrate transcriptome with N-deprived conditions and found a partial overlap of differentially regulated genes between these conditions. Nitric oxide (NO)-metabolizing flavohaemoglobins were found to be co-regulated with nitrate assimilatory genes. Subsequent molecular characterization revealed that the strongly inducible FhbA is required for full activity of nitrate and nitrite reductase enzymes. The co-regulation of NO-detoxifying and nitrate/nitrite assimilating systems may represent a conserved mechanism, which serves to neutralize nitrosative stress imposed by an external NO source in saprophytic and pathogenic fungi. Our analysis using membrane-permeable NO donors suggests that signalling for NirA activation only indirectly depends on the nitrate transporters NrtA (CrnA) and NrtB (CrnB).

## Introduction

Nitrate (NO_3_^-^) is the most abundant inorganic nitrogen (N) form in soils, and plants (Song *et al*., 2007), fungi (Joergensen and Wichern, 2008; Inselsbacher *et al*., 2009), bacteria (Klotz and Stein, 2008) and archaea (Erguder *et al*., 2009) all compete for this resource. Fungal biomass dominates in many soil types and although some fungi show denitrifying activities (Takaya, 2002; Fujii and Takaya, 2008; Kim *et al*., 2009) the presence of nitrate and nitrite assimilation genes in most fungal phyla (M. Gorfer and J. Strauss, unpubl. obs.) indicates a relevant ecological function of this metabolic system in soil nitrogen cycling. Provided sufficient carbon supply, fungi are able to efficiently utilize nitrate and nitrite for growth and incorporate this nitrogen source into biomass through reduction to ammonium and subsequent metabolization to glutamate and glutamine (Cove, 1979). Because of the high metabolic cost of nitrate reduction, negative feedback regulation by ammonium and glutamine prevents the expression of genes required for the uptake and assimilation of nitrate or nitrite (Caddick *et al*., 1994; Marzluf, 1997; Berger *et al*., 2008). Phenomenologically similar circuits also regulate nitrate assimilation in photosynthetic eukaryotes such as the algae *Chlamydomonas reinhardii* (Fernandez and Galvan, 2008) and plants (Crawford and Arst, 1993). However, because of additional constitutively expressed nitrate transporters, regulation of nitrate reductase (NR) by light and the function of nitrate as a developmental signal, nitrate regulation in plants is more complex (Daniel-Vedele *et al*., 1998; Zhang and Forde, 2000; Glass *et al*., 2002; Wang *et al*., 2003; Miller *et al*., 2007).

In *Aspergillus nidulans,* the transcriptional control of the assimilatory system is carried out by the synergistic action of the nitrate-specific activator NirA (Burger *et al*., 1991; Strauss *et al*., 1998) and the nitrogen status-sensing regulator AreA (Kudla *et al*., 1990). Nitrate and nitrite uptake is carried out via specific permeases (Brownlee and Arst, 1983; Unkles *et al*., 1991; 2001; Wang *et al*., 2008) and subsequently, intracellular nitrate mediates accumulation of NirA in the nucleus. We have previously shown that nitrate functions to disrupt the interaction between the NirA nuclear export signal and the KapK–NplA nuclear export complex (Bernreiter *et al*., 2007). However, nuclear accumulation is required, but not sufficient for NirA activity but the exact mechanism by which nitrate acts to transform NirA into an active transcription factor has not been elucidated so far. Interestingly, in the simultaneous presence of external nitrate and ammonium NirA remains nuclear, but does not efficiently bind DNA. *In vivo* DNA binding of NirA requires, in addition to nitrate, an active, DNA-bound form of AreA (Narendja *et al*., 2002). The two transcription factors physically interact (Muro-Pastor *et al*., 2004; Berger *et al*., 2008) recruit chromatin remodelling complexes (Muro-Pastor *et al*., 1999; Berger *et al*., 2008) and act synergistically to activate transcription. The recruitment of chromatin remodelling complexes to the nitrate locus by AreA can be artificially triggered by extended nitrogen starvation (Berger *et al*., 2008), a condition in which very high levels of AreA accumulate in the nucleus (Todd *et al*., 2005). AreA is not only required for expression of nitrate genes, but positively regulates, in most cases in cooperation with the respective pathway-specific transcription factor, a range of permeases and catabolic enzymes required for the utilization of alternative, metabolically ‘costly’ nitrogen sources such as nucleotides, amino acids or primary and secondary amides (Caddick *et al*., 1994; Scazzocchio, 2000 and references therein). Several positive and negative feedback mechanisms operate to ensure that AreA is only active when available nitrogen is limited. Under nitrogen limitation conditions, AreA abundance and activity increases because of higher *areA* gene transcription (Langdon *et al*., 1995) and mRNA stability (Morozov *et al*., 2000; 2001; 2010; Caddick *et al*., 2006), and a preferential nuclear localization of the protein (Todd *et al*., 2005). Nitrogen sufficiency reduces AreA abundance and the activity of the protein is reduced by interaction with the negative regulatory protein NmrA (Andrianopoulos *et al*., 1998; Wong *et al*., 2007).

At the genomic level, the response to nitrate has been extensively studied in plants. Roughly 10% of the *Arabidopsis thaliana* detectable transcriptome responds to nitrate supply (primary response) and includes upregulation of genes involved in nitrate transport and assimilation. Plant nitrate assimilation is strongly influenced by light and the circadian rhythm, and responds to the carbon status, as well as hormonal and developmental signals (reviewed in Krouk *et al*., 2010). Because of this complexity, the nitrate response has been found to be highly context-dependent (genotypes, slight differences in experimental design) as only about 300 genes were identified in a meta-analysis of transcriptome datasets from different laboratories to be consistently nitrate-regulated (Gutierrez *et al*., 2007). It has been hypothesized that the high variability between different datasets might reflect a complex gene network providing the necessary nutritional adaptability of sessile organisms to fluctuating environmental conditions. Despite the enormous progress made in understanding the interface between nitrogen and other regulatory sub-networks using Systems Biology approaches, little is still known on transcriptional regulators of plant nitrate assimilation genes. Several regulatory proteins have been identified as responding to NO_3_^-^, such as the MADS-box NAR1 protein controlling lateral root development (Zhang and Forde, 2000), several protein kinases regulating the uptake system (Ho *et al*., 2009; Hu *et al*., 2009), and NLP7, a nuclear protein with putative regulatory functions in nitrate signalling and the starvation response (Castaings *et al*., 2009). NLP7 is similar to the *C. reinhardii* NIT2 regulator, which was shown to be the pathway-specific activator for nitrate assimilation genes in this unicellular algae (Camargo *et al*., 2007). Initial protein–DNA interaction studies in the tobacco NR gene promoter showed a nitrate-inducible protection of GATA sites (Rastogi *et al*., 1997) reminiscent of AreA binding in *Aspergillus* nitrate-inducible genes (Muro-Pastor *et al*., 1999; Berger *et al*., 2008). Unfortunately, the involvement of putative plant GATA factors in nitrogen regulation was not substantiated by further studies leaving the nature of plant nitrate assimilation regulators still uncertain.

The work presented here shows that the fungal nitrate response reprograms about 1% of the *A. nidulans* transcriptome including genes for nitrate transport and metabolism. Several novel genes coding for putative regulators of the nitrate response were uncovered and we also found an inducible nitric oxide-detoxifying flavohaemoglobin (FhbA) as target of the pathway-specific regulator NirA. Characterization of *fhb* genes provided evidence for an important physiological role of the enzymes under a variety of environmental conditions. Results using membrane-permeable NO donors suggest that signalling for NirA activation only indirectly depends on the nitrate transporters NrtA/CrnA and NrtB/CrnB.

## Results

### Experimental set-up and physiological responses to nitrate induction and N-starvation

**Fig. 1:**
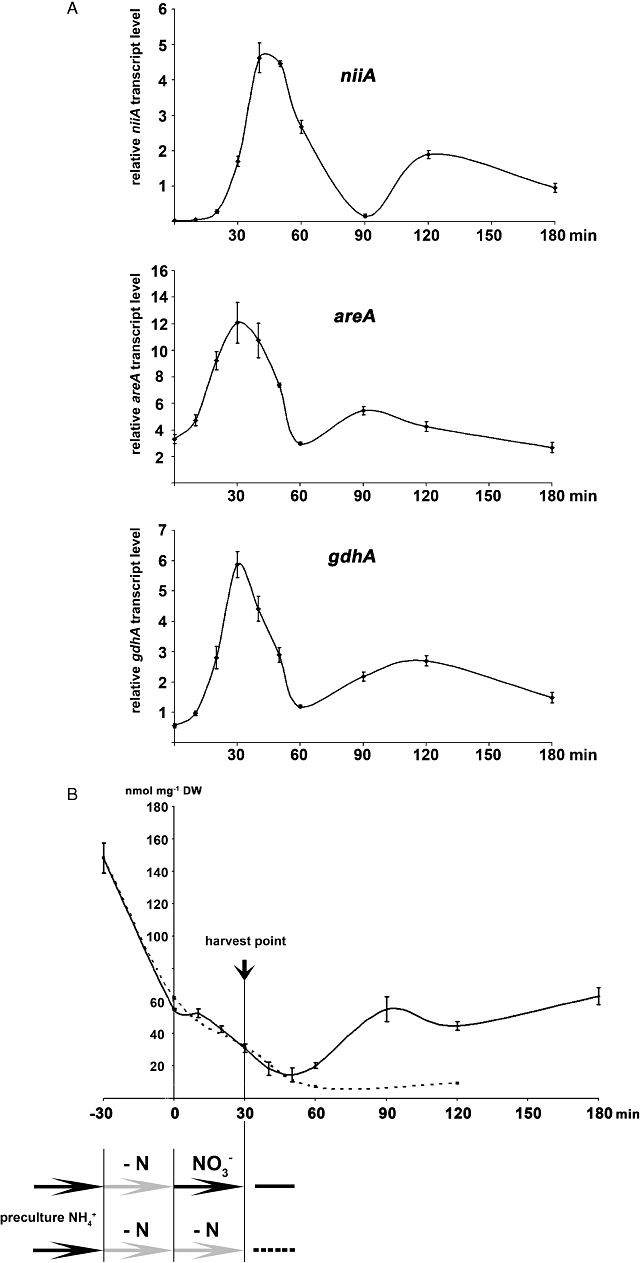
A. Transcriptional profile of marker genes *niiA* (nitrite reductase), *areA* (GATA TF) and *gdhA* (NADPH-glutamate dehydrogenase) during the nitrate induction process in the wild-type strain. mRNA levels were determined by RT-qPCR (see *Experimental procedures*). The normalized levels of specific transcripts are shown relative to *acnA* (actin) over a time period of 180 min. Aliquots were taken from the cultures at the induction starting point (0 min) and then after 10, 20, 30 40, 50, 60, 90, 120 and 180 min following induction by 10 mM NaNO_3_. Standard deviations are calculated from three independent biological replicates.B. Schematic representation of experimental design and measurements of intracellular free amino acid levels. Cultures were grown in glucose minimal medium (GMM) in the presence of 10 mM NH_4_^+^ as the sole N-source for 14 h (preculture NH_4_), harvested, washed, resuspended in GMM lacking any nitrogen source and incubated under these conditions for 30 min (−N). Wild-type and *nirA*^-^ cells subsequently received 10 mM NaNO_3_ (NO_3_), whereas starved wild-type cells were further incubated without nitrogen addition (−N). Incubation proceeded for another 30 min until they were harvested for the microarray experiment (harvest point). For qPCR analysis and amino acid level determination cells were further incubated until 180 min. The graph shows intracellular free glutamine (Gln) levels measured immediately before the starvation period (−30), at the end of the starvation period (0) and then subsequently after 10, 20, 30, 40, 50, 60, 90, 120 and 180 min of NO_3_^-^ induction (full lines) or after 30, 60, 90 and 150 min of continuous starvation (dotted lines) in wild-type cells. Values on the *y*-axis represent nmoles free Gln mg^−1^ dry weight (DW), standard deviations are from three independent experiments.

Our interest was to identify *A. nidulans* genes that showed a short-term response to the presence of nitrate in the growth medium (induced conditions), as compared with growth conditions in which ammonium was supplied to the cultures (repressed conditions). In order to limit indirect effects from downstream metabolites of nitrate assimilation, i.e. the formation of repressing nitrogen metabolites during continuous nitrate assimilation, we first performed a time series of nitrate induction. In these cultures we monitored the expression of marker genes, which are known to sensitively respond to the intracellular nitrogen status (Fig. 1A) and free amino acid pools (Fig. 1B and Table S1). Maximum *niiA* mRNA levels were reached after 40 and 50 min of induction, indicating maximum synergistic NirA–AreA activity. Maximum *niiA* levels coincided with a minimum of intracellular glutamine concentration. *areA* and *gdhA* levels responded roughly 20 min earlier and reached maximum transcript levels after 30 min. Up until 50 min of nitrate induction intracellular Gln levels continued to decline despite fully induced NR enzyme levels (Fig. S1). This indicates that the low Gln levels found at this time point are the result of increasing metabolic activity, which creates a higher demand for amino acids. In agreement with our previous results (Berger *et al*., 2008) Gln, but not Glu, appears to serve as intracellular amino acid storage and signalling pool (Fig. S2). Continuous NO_3_^-^ assimilation (starting from 50 min onwards) produced more Gln than was utilized by metabolic activities and this led to a replenishment of the free Gln pool (Fig. 1B). Consequently, higher Gln levels led to partial AreA inactivation and thus to reduced transcriptional activity of the NirA–AreA activator complex and to a drop in amounts of *niiA* mRNA (Fig. 1A). Interestingly, *areA* and *gdhA* mRNA levels were continuously decreasing between 30 and 60 min of nitrate induction although the lowest levels of Gln were found at 50 min. This indicates that transcription and/or mRNA stability of *areA* and *gdhA* responds to additional metabolic signals related to the intracellular nitrogen or carbon status. The exact nature of such signals remains to be identified.

### Growth conditions for transcriptome analysis

As a consequence of the time series experiments, we performed our transcriptome analysis with RNA derived from 30 min induced cultures because the low free Gln pool would prevent negative interference of assimilation products with the induction process. The 30 min induced cultures were compared with fully repressed cultures (e1 = WT^NH^_4__vs_WT^NO^_3_^-^), i.e. cells that were transferred after pre-growth on ammonium again to ammonium-containing medium without an interim starvation period. In order to test which of the nitrate-regulated genes are dependent on the pathway-specific activator NirA, we performed an identical set of induction experiments with a *nirA*637 loss-of-function strain (e3 = *nirA*637^NH^_4__vs_*nirA*637^NO^_3_^-^). It is important to note that nitrate uptake is only slightly impaired in *nirA*^-^ strains (Brownlee and Arst, 1983). This experiment allowed us to differentiate between nitrate-regulated genes that are a direct target of NirA and genes, which respond to nitrate induction independently of the transcription factor.

Physiologically, nitrate induction in a *nirA* loss-of-function strain is equivalent to strong nitrogen limitation, because the NR activities found during the induction period in the wild-type strain were reduced to very low levels in the *nirA*637 mutant (Fig. S1). Therefore, we also performed transcriptome analysis of wild-type cells subjected to continuous nitrogen starvation by incubation on nitrogen-free medium (−N conditions) for 60 min and compared this transcriptome with cells continuously grown on ammonium (e2 = WT^NH^_4_^+^_vs_WT^−N^). We reasoned that these conditions would enable us to differentiate between genes responding to nitrogen limitation and genes responding to nitrate in the absence of the transcription factor.

### Overview of differentially expressed genes

**Fig. 2:**
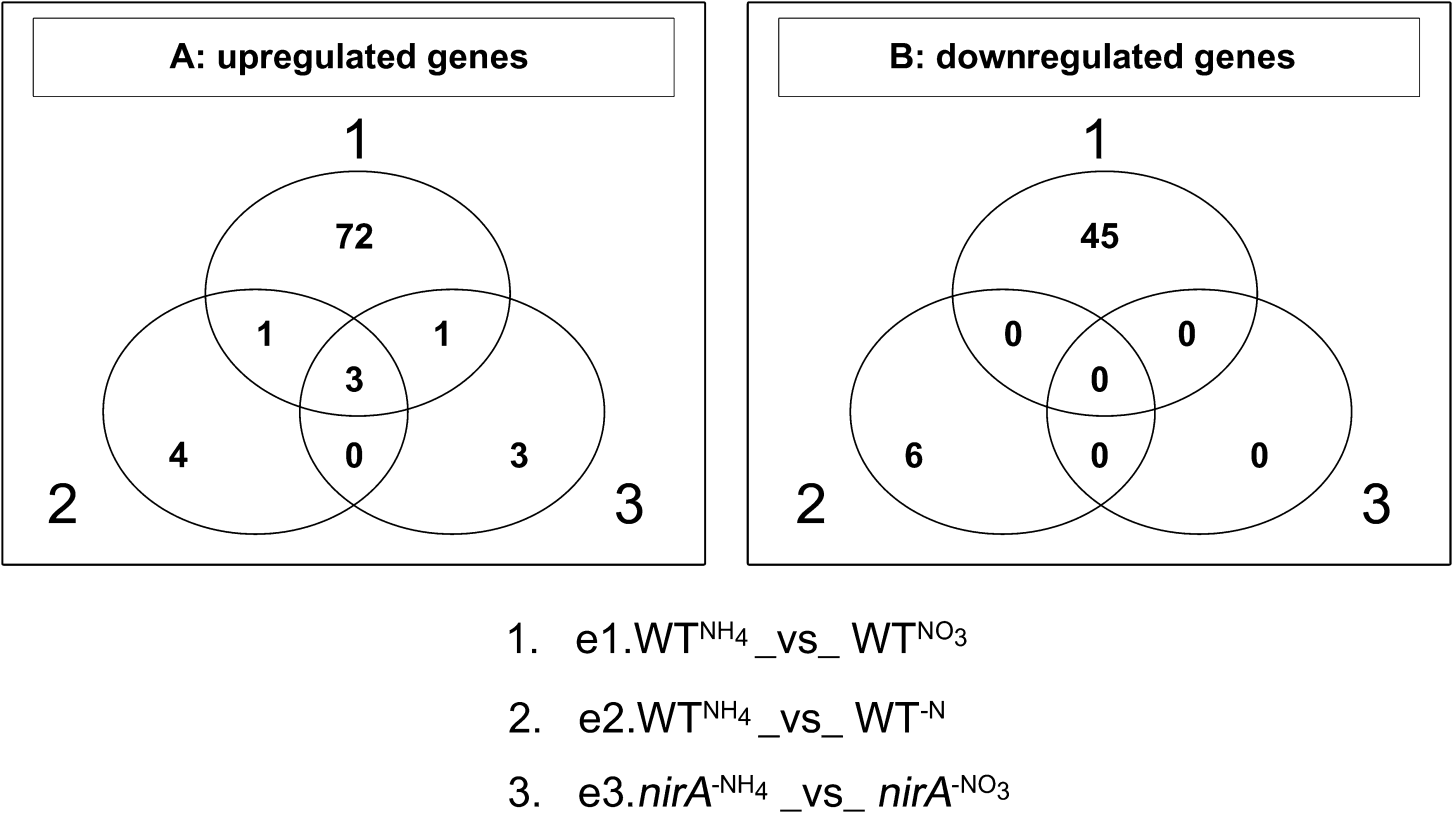
Differentially expressed genes in the nitrate and starvation response. The Venn diagram shows genes at least fourfold (log_2_ ≥ 2) upregulated (A) or downregulated (B) in response to different treatments. Numbers in the circles represent numbers of responding genes and numbers in overlapping sections represent genes that are commonly regulated by two or three conditions and/or in different strains. Circle 1 represents genes differentially regulated by NO_3_^-^ induction in the wild type (experiment e1. WT^NH4^_vs_WT^NO3^), circle 2 represents genes regulated by nitrogen depletion in wild-type cells (experiment e2. WT^NH4^_vs_WT^−N^), whereas circle 3 represents genes of NO_3_^-^-treated *nirA*^-^ cells that can not metabolize this nitrogen source (experiment e3. *nirA*^−NH4^_vs_*nirA*^−NO3^).

Analysis of the microarray data revealed a total of 135 differentially expressed genes (DEGs) for all three experimental set-ups when thresholds of fourfold change (log_2_ ≥ 2) and a *t*-test value of *P* ≤ 0.05 were applied (see *Experimental procedures* for details). Eighty-four genes responded to nitrate induction or nitrogen starvation by upregulation whereas 51 genes showed lower expression under these conditions. The Venn diagram in Fig. 2 shows a synopsis of DEGs from all experiments. Among the genes upregulated by at least fourfold 76 genes responded to NO_3_^-^ (72 in WT and four in *nirA*^-^) whereas four genes were also upregulated by N-limitation (one in WT^−N^ and three in *nirA*^−NO^_3_^-^). An additional four genes positively responded to N-limitation in the wild type and three genes showed upregulation only in NO_3_^-^-induced cultures in *nirA*^-^ strains. Also surprising is the comparatively low number of genes responding by at least fourfold upregulation to the limited time of nitrogen starvation (60 min total) applied in this experiment. The regulatory response during N-starvation (compare Tables S3 and S4) seemed to be less strong (5- to 10-fold upregulation), compared with the nitrate induction response (up to 60-fold upregulation). It should be noted, however, that our experimental conditions most probably do not represent full nitrogen starvation. A much more pronounced change in global gene transcription could be expected in this case, similar to what has been observed in other fungi (Matsuo *et al*., 2007; Schonig *et al*., 2008; Staschke *et al*., 2010).

A considerably high number of genes (45 DEGs) were downregulated in response to NO_3_^-^ induction in the wild type. This negative response depended on NirA function and/or the nitrate assimilation process because no downregulated gene was detected in *nirA*637 under NO_3_^-^ conditions. N-limited cells (WT-N) did not respond strongly by downregulation: only six genes were repressed by at least fourfold, but as in this case, like in upregulated DEGs, the limited time of N-deprivation might have restricted the response.

### The function of NirA in the induction process

A vast majority (72 genes) of the total 80 genes positively responding to NO_3_^-^ induction in e1 (WT) and e3 (*nirA*^-^) required the function of NirA. The central importance of NirA in the induction process is evident from the Venn diagram (Fig. 2) and functional assignments (Table 1 and Table S2), which shows that only seven genes responded to NO_3_^-^ in the *nirA*^-^ mutant and three of these genes overlapped with the group of starvation-induced genes, making 95% of DEGs NirA-dependent for NO_3_^-^ induction. NirA requirement could be either direct, i.e. promoters carry functional 5′CTCCGHGG consensus target binding sites for NirA, or indirect, i.e. the upregulation response depends on nitrate or its metabolism. Bioinformatic analysis of 1 kb upstream regions of the 72 open reading frames revealed 16 promoters, which carry either confirmed or putative NirA binding sites (Table S3). Thus, for 51 NO_3_^-^- and NirA-dependent genes upregulation was indirect, most probably depending on the active nitrate assimilation process in which the transcriptionally active NirA–AreA complex would trigger secondary transcriptional activation events. In contrast, only seven genes positively responded to nitrate in a *nirA*637 loss-of-function mutant. Three of those genes are identical to genes responding to nitrogen starvation and thus are genes not requiring NO_3_^-^ for upregulation. Only one gene was commonly upregulated by NO_3_^-^ (but not under −N conditions) in the *nirA*^+^ and *nirA*^-^ strain and therefore should be classified as a gene directly responding to the presence of NO_3_^-^ in the cell. Interestingly, three genes were upregulated by NO_3_^-^ only when NirA is not functional suggesting a negative role for NirA and/or NO_3_^-^ metabolism in the transcriptional activation process of these genes. Because none of the three genes carries predicted NirA binding targets in their 1 kb upstream region, the negative function of NirA is most likely indirect.

**Table 1:** Summary of DEGs in different functional categories.

Main categories^a^	NO_3_-induced WT	NO_3_-repressed WT	−N-induced WT, *nirA*^-^	−N-repress. WT, *nirA*^-^
Total number of genes	72	45	12	6
NO_3_ metabolism, direct NirA targets^b^	16	0	0	0
NirA targets, known functions	6	0	0	0
NirA targets, predicted or unknown function	10	0	0	0
Regulatory role^c^	8	8	2	2
Amino acid metabolism^c^	11	0	8	3
Carbon metabolism^c^	5	5	1	0
Lipid/secondary metabolism^c^	11	6	0	1
Hypothetical proteins, no known function^c^	31	25	1	0

a Genes are grouped according to major metabolic categories, including a subcategory for genes putatively playing a role in nitrate assimilation. Genes can be grouped into more than one subcategory.

b Classification based on gene regulation (only induced in WT) and known or putative NirA binding consensus sites present in the 1 kb upstream region.

c Function in the metabolic pathways could be known or predicted based on similarity to proteins with known function.

### Established and putative functions of genes positively responding to NO_3_^-^

Among the genes that most strongly responded to nitrate by 15- to 60-fold upregulation are the extensively characterized genes known to be involved in the nitrate assimilation process, such as *niaD* and *niiA*[coding for NR and nitrite reductase (NiR) respectively], the two nitrate transporter genes *crnA/nrtA* and *crnB/nrtB*, and the nitrite transporter gene *nitA*. A gene strongly upregulated (12-fold increase) but so far not known to respond to nitrate induction encodes a putative flavohaemoglobin (AN07169.1). This family of proteins is known to function in nitric oxide (NO) detoxification, converting the short-lived NO radical to nitrate (Gardner *et al*., 1998; Foster *et al*., 2009). The *A. nidulans* genome shows two highly conserved putative flavohaemoglobins and based on the known function of NO in signalling events, we studied these genes in more detail (see below). It is noteworthy that genes with a putative involvement in morphology determination and cytoskeleton function (putative chitin synthethase III, AN4367.3; putative hydrophobin gene AN7539.3; putative fluffy-determinant gene AN9451.3;) were upregulated by NO_3_^-^ induction, although these genes do not carry predicted NirA binding sites in their 5′UTR and thus the response is likely to be a consequence of the nitrate assimilation process. (Table S3). Several other novel genes that positively responded to nitrate encode proteins with a predicted function in the mobilization of extracellular and internal nitrogen sources. For example, we find genes coding for proteases and genes involved in the metabolism and transport of nitrogen-containing compounds such as amino acids, urea, allantoin and nucleotides (see Table S3). Our experimental set-up also revealed the activation of genes coding for putative signalling and regulatory proteins, such as kinases, GTP-binding proteins, guanylate kinase, helicase and transcriptional activators and repressors. Leucine biosynthesis and acetyl-coenzyme-A metabolism seem to occupy a special position in nitrate assimilation. Hypothetical isopropylmalate dehydrogenase (AN2793) and isomerase (AN5886) as well as dihydroxy-acid dehydratase (AN7358) genes are proposed to be involved in leucine biosynthesis and are upregulated by NO_3_^-^. As they do not contain predicted NirA binding sites within 1 kb of their 5′regions, the induction is indirect through the NO_3_^-^ assimilation process. Acetyl-coA metabolism (putative d-lactate dehydrogenase AN0628; enoyl-coA hydratase AN0180) and connected lipid metabolic genes (putative mitochondrial malic enzyme AN6933; lipase AN2602; esterase AN2834) also appear to be an overrepresented category. As two of these genes contain predicted NirA binding sites in their promoters (AN2834 and AN0628) the induction response might be directly performed by NirA. The connection of NO_3_^-^ and leucine/coenzyme-A metabolism might provide a link to secondary metabolism. It is known that NO_3_^-^ stimulates production of sterigmatocystin and penicillin in *A. nidulans* (Brakhage, 1998; Feng and Leonard, 1998) and acetyl-coA is a crucial precursor for the vast majority of fungal secondary metabolites including antibiotics (Keller *et al*., 2005). The elevated transcription of genes responsible for acetyl-coA, isopropylmalate and l-leucine biosynthesis (pathways connected to the penicillin precursors valine and α-l-aminoadipic acid) would be a logical hypothesis to explain the stronger production of secondary metabolites on nitrate as sole N-source in *A. nidulans*.

### Genes positively responding to nitrogen limitation

Under the conditions (1 h nitrogen limitation) and thresholds (at least fourfold upregulation) applied in this study, only 12 genes were significantly upregulated by nitrogen limitation (Table 1 and Table S4). Four of these genes overlapped with the group of nitrate-induced DEGs and thus upregulation was most likely directly related to the intracellular glutamine level, which was basically identical between the nitrate-induced and the N-starved conditions. A strong response was obtained for genes involved in ammonium uptake and metabolism: *meaA,* the low-affinity transporter (AN7463), *gdhA*, the NADPH-dependent glutamate dehydrogenase (AN4376) and *glnA*, glutamine synthethase (AN4159). Also the recently confirmed major urea transporter similar to Dur3 (AN0418) (Abreu *et al*., 2010) responded positively to nitrogen limitation. The remaining four DEGs, which code for a peptide transporter (AN8903), a putative dipeptidyl-peptidase (AN2572), a putative Zn-cluster transcription factor (AN1927) and a conserved hypothetical gene (AN1404) did not overlap with the NO_3_^-^-induced group despite the fact that identical Gln levels were observed between these two conditions. We hypothesize that these four latter genes respond to additional signals, e.g. low metabolic activities. In this context it is interesting to note that concentrations of some free amino acids were significantly higher after 1 h starvation than after 30 min NO_3_^-^ induction. This might be due to re-assuming metabolic activity and demand for amino acids in the NO_3_^-^-induced cells, compared with lower activities in the N-starved cells. Table S1 shows a comparison of amino acid concentrations in the wild type between these two conditions and for Asp, Asn, Arg, Ala and Tyr the values in NO_3_^-^-induced cells were significantly lower than in the metabolically less active cells incubated under N-limitation.

### Genes negatively responding to NO_3_^-^ and N-limitation

Addition of NO_3_^-^ resulted in downregulation of 45 genes (Table S5). None of these genes appeared to be repressed in the *nirA*^-^ strain suggesting that the function of NirA is required directly or indirectly for this negative response. Only one out of the 45 genes, i.e. AN10059, which putatively encodes a fungal-specific C6 transcription factor, harbours a consensus NirA binding site. Therefore, all the other 44 genes most likely responded to the active nitrate assimilation process. The majority of repressed genes (25 genes) encode hypothetical or conserved hypothetical proteins with no predicted functions. As shown in Table S5, five genes encode proteins with similarities to enzymes involved in the breakdown of complex carbon sources (α- and β-galactosidase, mannanase and rhamnogalacturan-lyase) and the products of eight of these genes show similarities to proteins, which regulate cellular metabolism (e.g. putative signalling kinase, mRNA stability regulation, transcription of ribosomal genes).

Not overlapping with the NO_3_^-^-repressed genes was a group of six genes that were downregulated by 60 min N-limitation (Table S6). These genes putatively encode proteins functioning in diverse cellular processes with no preference to a specific pathway.

### Flavohaemoglobin *fhbA* is co-regulated with the nitrate assimilation system

**Fig. 3:**
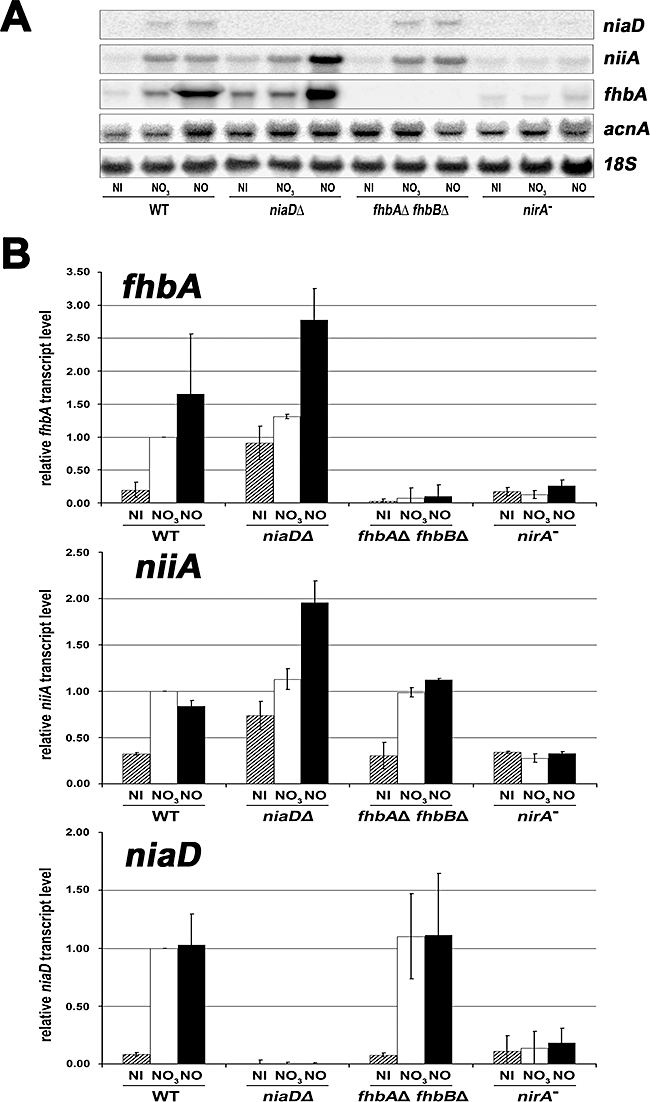
Northern blot analysis (A) and quantification of expression levels (B) of nitrate reductase (*niaD*), nitrite reductase (*niiA*) and flavohaemoglobin A (*fhbA*). Actin (*acnA*) and 18*S* rRNA (18*S*) served as loading controls. Wild type (WT) was compared with deletion mutants in nitrate reductase (*niaD*Δ), and flavohaemoglobins A and B (*fhbA*Δ*fhbB*Δ), and with a *nirA*637 loss-of-function mutant (*nirA*^-^). Strains were cultivated as described in *Experimental procedures* on 3 mM arginine (non-inducing, NI), 10 mM NO_3_^-^ (inducing, NO_3_) or 3 mM arginine and 1.5 mM DetaNONOate (NO-inducing, NO). ^32^P signals were analysed by phosphoimaging and expression levels in (B) are expressed as percentage of induced wild type, which was arbitrarily set to a value of 1. Data of two independent experiments were combined and standard deviations were calculated.

To gain further insight into the role of flavohaemoglobins during nitrate assimilation we studied these genes in more detail. The NO_3_^-^-inducible gene is most similar to yeast YHB1 and *Cryptococcus neoformans* FHB1 and was designated *fhbA* according to the *A. nidulans* nomenclature. The second putative flavohaemoglobin gene (designated *fhbB*) was only twofold induced by nitrate (data not shown) and the predicted protein carries a putative mitochondrial target sequence similar to *fhbB* in *Aspergillus oryzae* (Zhou *et al*., 2009). Both genes were deleted individually and in combination in order to study their function during the NO_3_^-^ induction process. Northern analysis (Fig. 3) of *fhbA* confirmed microarray data and showed that *fhbA* is co-regulated with the main assimilatory genes such as *niaD* and *niiA*. Strong *nirA*-dependent induction by NO_3_^-^ conditions and high constitutive transcript levels in a *niaD*Δ background (Cove and Pateman, 1969; Hawker *et al*., 1992) were observed for *fhbA*.

### FhbA expression is AreA-independent

**Fig. 4:**
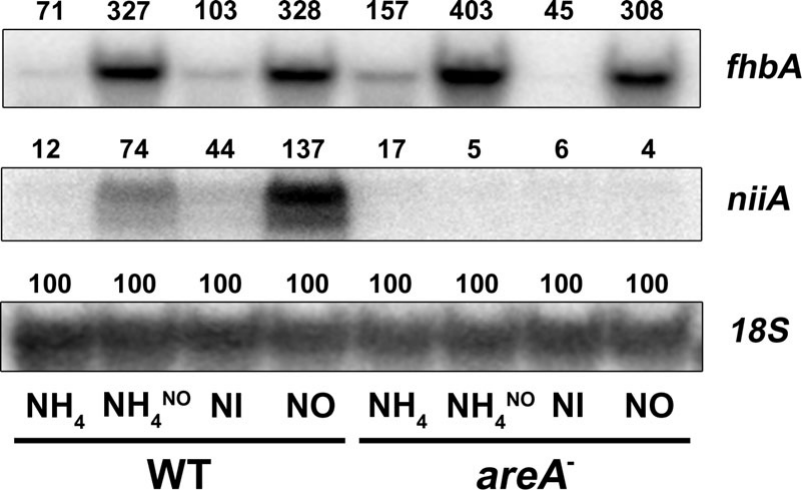
Northern blot analysis of flavohaemoglobinA (*fhbA*), nitrite reductase (*niiA*) and 18S rRNA (loading control) transcript levels. Wild type (WT) and *areA*600 loss-of-function mutant (*areA*^-^) were cultivated under different nitrogen conditions as described in *Experimental procedures* under repressed (NH_4_); NO-induced and repressed (NH_4_^NO^), non-induced (NI); and NO-induced (NO) conditions. Northern blots were probed with ^32^P-labelled *niiA* and *fhbA* probes and signals were analysed by phosphoimaging. Numbers below the signals indicate relative expression levels in comparison with 18S rRNA, which was arbitrarily set to 100%.

We tested if *fhbA* is regulated as other known nitrate-responsive genes, i.e. induced by NO_3_^-^ and subject to nitrogen metabolite repression by external ammonium (NH_4_^+^) mediated through the inactivation of the co-activator AreA. Figures 3 and 4 also show the expression profile of the *niiA* gene used as internal control. *niiA* was strongly induced by NO_3_^-^ and NO, dependent on AreA function, and subject to nitrogen metabolite repression when NH_4_^+^ was added to the medium simultaneously with NO. In contrast, expression of *fhbA* was independent of AreA, showing no ammonium-induced repression in the presence of NO and even a slight constitutive level under fully repressing conditions (NH_4_^+^ in WT and *areA*^-^). This is a surprising result, as all genes known so far to be regulated by NirA depend also on functional AreA. Moreover, four AreA consensus binding sites (5′WGATAR) are present within 500 bp of the *fhbA* promoter, in addition to a perfectly matching NirA site at position −225 from the ATG start codon. This sequence arrangement would suggest a canonical NirA–AreA synergism in *fhbA* activation although this is not the case. There are at least five other transcriptional regulators known in *A. nidulans* to contain functional GATA-type DNA-binding domains and some of these proteins, such as the iron-responsive repressor SreA (Oberegger *et al*., 2001) or the light-responsive regulators of sexual development LreA and LreB (Purschwitz *et al*., 2008), might bind to the *fhbA* promoter and participate in transcriptional regulation of this gene.

### The role of NO in the regulation process

**Fig. 5:**
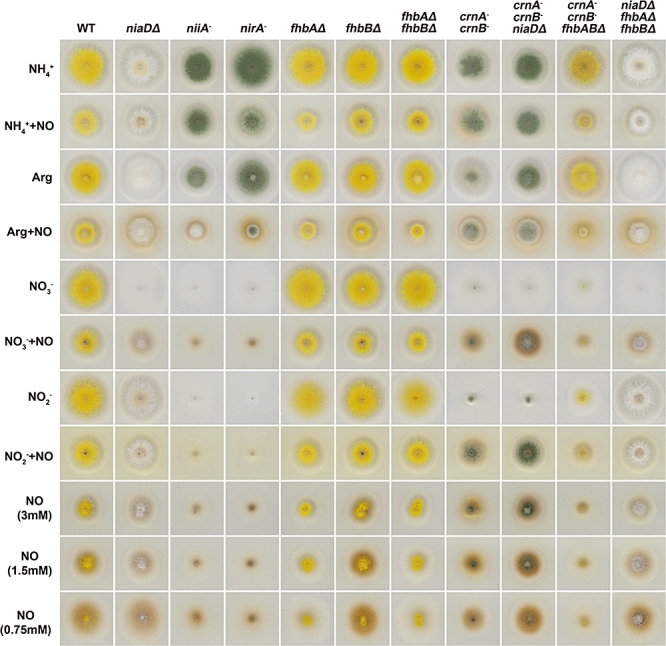
Growth test on solid glucose minimal media at pH 6.8, and supplements according to strain requirements. Plates were prepared containing the following N-sources: 10 mM ammonium (NH_4_), 10 mM ammonium + 1.5 mM DetaNONOate (NH_4_ + NO), 3 mM arginine (Arg), 3 mM arginine + 1.5 mM DetaNONOate (Arg + NO), 10 mM nitrate (NO_3_), 10 mM nitrate + 1.5 mM DetaNONOate (NO_3_ + NO), 10 mM nitrite (NO_2_), nitrite + 1.5 mM DetaNONOate (NO_2_ + NO) and different nitric oxide (NO) concentrations (0.75, 1.5 and 3 mM). *A. nidulans* wild-type strain (WT), nitrate reductase mutant (*niaD*Δ), nitrite reductase mutant *niiA*4 (*niiA*^-^), *nirA*637 mutant (*nirA*^-^), single and double flavohaemoglobin mutants (*fhbA*Δ; *fhbB*Δ; *fhbA*Δ*fhbB*Δ) and nitrate transporter deficient strains (*crnA*^-^*crnB*^-^) were used in the growth experiment. After 72 h at 37°C growth was documented by light scanning and the figure was composed using Photoshop 8.0.

**Fig. 6:**
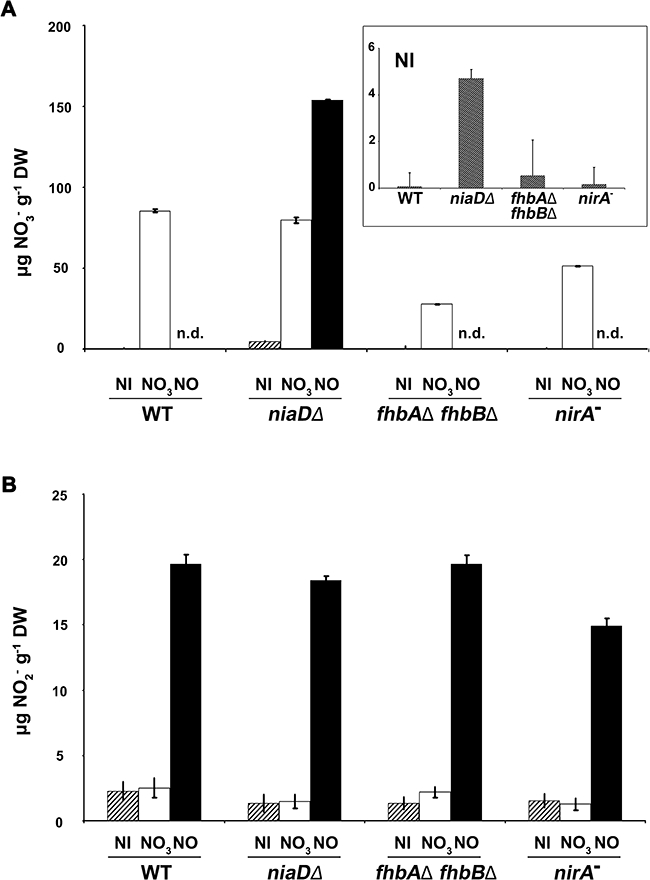
Intracellular nitrate (A) and nitrite (B) levels on different nitrogen sources. The levels were measured according to the method described in *Experimental procedures* in *A. nidulans* wild-type cells (WT), and in mutants lacking nitrate reductase (*niaD*Δ), both flavohaemoglobin genes (*fhbA*Δ*fhbB*Δ), or functional NirA (*nirA*^-^). As for Northern analysis, strains were pre-grown on arginine as a non-inducing nitrogen source and harvested mycelia N-starved prior to shift to media containing different nitrogen sources: 3 mM arginine (NI, shaded bars); nitrate-inducing conditions with 3 mM arginine + 10 mM nitrate (NO_3_, white bars); or nitric oxide-inducing conditions with, 3 mM arginine + 1.5 mM DetaNONOate (NO, filled bars). NO_3_^-^ values under NI conditions are shown in the magnified insert panel. Data of a representative experiment, reflecting the observed overall pattern, are shown and the amounts and calculated standard deviations of NO_3_^-^ and NO_2_^-^ are expressed as µg per gram dry weight (µg g^−1^ DW). n.d. denotes non-detectable amounts of NO_3_^-^ (for details see Supplementary Experimental procedures).

Treatment of the cells with the synthetic NO donor DetaNONOate led to a strong upregulation of all tested nitrate assimilation genes. The transcriptional response to NO was not dependent on FhbA or FhbB function, i.e. occurring equally well in the *fhbA*Δ*fhbB*Δ double mutant, making it unlikely that the induction by NO is only a consequence of NO_3_^-^ formation by flavohaemoglobin activities. DetaNONOate induction could also be triggered by formation of NO_2_^-^, which arises spontaneously from NO oxidation in the medium and inside the fungal cell (Yamasaki, 2000). To differentiate effects of NO and NO_2_^-^, we measured the intracellular concentrations of NO (Fig. S3) by electron paramagnetic resonance (EPR) spectroscopy (Xu *et al*., 2005; Pirker *et al*., 2008) and of NO_2_^-^ and NO_3_^-^ (Fig. 6) by a modified colorimetric assay (Inselsbacher *et al*., 2009). EPR is based on the trapping of NO radicals by the spin trap Fe^2+^-diethyldithiocarbamate (Fe^2+^-DETC) forming a Fe^2+^-DETC–NO complex. This complex is detected as triplet in the EPR spectrum, which arises from the interaction of the unpaired electron with a nitrogen nucleus (spin I = 1) in the complex. Additionally, growth tests were performed (Fig. 5) using strains, which lack or have strongly reduced NO_3_^-^ and NO_2_^-^ transport respectively (Unkles *et al*., 2001; Wang *et al*., 2008). The growth tests (Fig. 5) showed that the transporter double mutant (lane *crnA*^-^*crnB*^-^) is impaired in NO_3_^-^ and NO_2_^-^ utilization leading to a strongly reduced growth on these N-sources. The same strain grown on nitrate or nitrite plus NO (rows NO_3_^-^ + NO and NO_2_^-^ + NO) showed significantly improved growth, presumably because of formation of NO_3_^-^ by flavohaemoglobins and of NO_2_^-^ by intracellular spontaneous oxidation. The amine-containing Deta-moiety (diethylene-triamine) of DetaNONOate did not serve as a relevant nitrogen source in these growth tests as both *niiA*^-^ and *nirA*^-^ strains showed very poor growth on all DetaNONOate concentrations tested. This indicates that the extracellular and intracellular conversion of NO to NO_2_^-^ and the intracellular generation of NO_3_^-^ by FhbA and FhbB are the responsible mechanisms for DetaNONOate utilization as sole N-source. EPR measurements to determine intracellular NO levels (Fig. S3) showed that in all NO-treated cells high amounts of this free radical were captured. Interestingly, we also detected low levels of NO in wild-type and *fhbAB* deletion mutants grown on nitrate. These data indicate that NO_3_^-^ assimilation in *A. nidulans,* similar to the situation reported in plants, results in the formation of NO and provides a physiological link to the observed co-regulation of assimilatory genes with flavohaemoglobins.

When we measured intracellular NO_2_^-^ levels in NO-treated cells (Fig. 6B) we found about 20 µg NO_2_^-^ per gram dry weight (g^−1^ DW). This was a roughly 10-fold higher NO_2_^-^ concentration compared with the one found in actively nitrate-reducing cells. Neither Fhb nor NR activities were needed for the conversion of NO to NO_2_^-^, consistent with a spontaneous intracellular NO oxidation. Intracellular NO_3_^-^ levels measured in NO-treated cells (Fig. 6A) confirmed the *in vivo* function of Fhb enzymes in the conversion of NO to NO_3_^-^ because the *fhbA*Δ*fhbB*Δ strain showed no detectable (n.d.) NO_3_^-^ accumulation after NO treatment. Surprisingly, the wild-type and *nirA*^-^ strains also showed no detectable intracellular NO_3_^-^ accumulation after DetaNONOate treatment. The simplest explanation consistent with these results is that, in the wild type, the simultaneously induced NR would effectively reduce the Fhb-generated NO_3_^-^ to NO_2_^-^. Indeed, a control strain lacking NR activity (*niaD*Δ) showed very high NO_3_^-^ levels after NO addition whereas in *nirA*^-^ strains, lacking *niaD* and *fhb* expression, NO conversion into NO_3_^-^ and subsequently accumulation, was not observed. Interestingly, the basal level expression of putative FhbB did not result in sufficient activity that would allow the detection of NO_3_^-^ in a *nirA*^-^ strain. In contrast, a *niaD*Δ strain showed clearly detectable NO_3_^-^ levels under non-induced (arginine) growth conditions (see insert panel in Fig. 6A) and these nitrate levels might be sufficiently high to promote expression of NirA target genes (*niiA*, *fhbA*) under non-induced conditions.

**Fig. 7:**
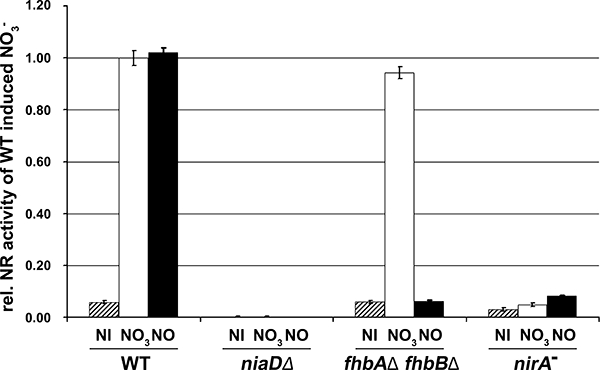
Nitrate reductase (NR) activity was measured in *A. nidulans* wild type (WT), and in mutants lacking nitrate reductase (*niaD*Δ), both flavohaemoglobin genes (*fhbA*Δ*fhbB*Δ), or NirA function (*nirA*^-^). Strains were pre-grown on arginine as non-inducing nitrogen source and harvested mycelia were starved prior to shift to media containing different nitrogen sources: 3 mM arginine (NI, shaded bars); nitrate-inducing conditions with 3 mM arginine + 10 mM nitrate (NO_3_, white bars); or nitric oxide-inducing conditions with, 3 mM arginine + 1.5 mM DetaNONOate (NO, black bars). Data of two independent biological experiments were merged and the activities of the wild-type NR under nitrate-inducing conditions were arbitrarily given a value of 1 (100%). Bars and respective standard deviations show NR activities relative to wild type under NO_3_^-^-induced conditions.

During these experiments we noted that the *fhb* double deletion strain displayed considerably lowered intracellular NO_3_^-^ levels (25% of WT) when grown on NO_3_^-^ (Fig. 6A). This could be the consequence of reduced NO_3_^-^ transporters function similar to the *nirA*^-^ strain (50% of WT) or could be due to increased NR activities. We therefore analysed NR activities under all relevant conditions (Fig. 7). We found that NR activities were not changed in *fhb* double mutants during growth on NO_3_^-^ but were drastically reduced in the mutants when DetaNONOate was added to the medium. These results provide evidence for an important role of flavohaemoglobins in protecting NR activity from damage directly by the NO radicals or indirectly by NO-derived reactive nitrogen species. This regulatory role of flavohaemoglobins seems to be relevant only at high concentrations of NO, as *fhbA*Δ single and *fhbA*Δ*fhbB*Δ double mutants grew normally on NO_3_^-^ as the sole N-source (Fig. 5). In contrast, stronger growth suppression was seen in *fhb* mutants compared with the wild type on NO_3_^-^ + NO, consistent with considerably reduced NR activities in the presence of the NO donor DetaNONOate (Fig. 7).

### A role for flavohaemoglobins in *Aspergillus* natural environments?

**Fig. 8:**
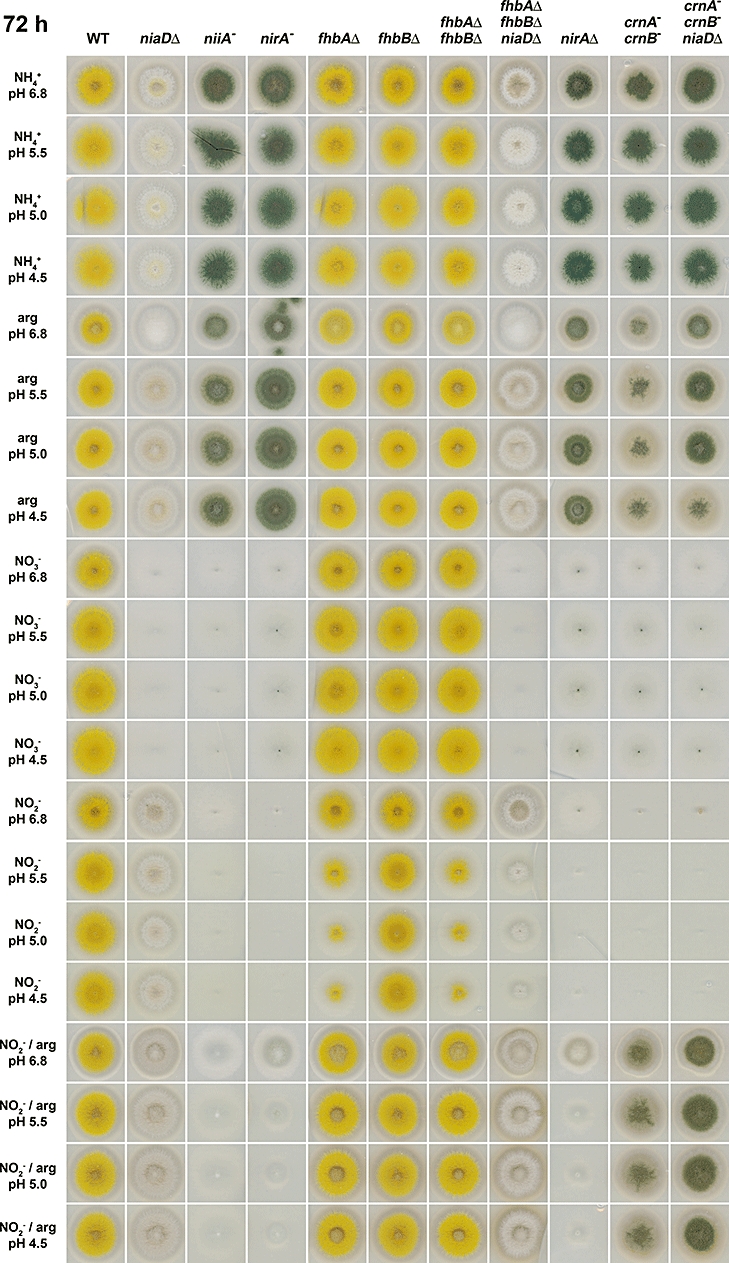
Growth test at different pH values on solid minimal media supplemented according to strain requirements. Plates were prepared containing the following N-sources: 10 mM ammonium (NH_4_), 3 mM arginine (Arg), 10 mM nitrate (NO_3_) or nitrite (NO_2_), or 10 mM nitrite + 3 mM arginine (NO_2_/arg). *A. nidulans* wild-type strain (WT), nitrate reductase mutant (*niaD*Δ), nitrite reductase mutant *niiA*4 (*niiA*^-^), *nirA*637 mutant (*nirA*^-^), single and double flavohaemoglobin mutants (*fhbA*Δ; *fhbB*Δ; *fhbA*Δ*fhbB*Δ), nitrate transporter deficient strains (*crnA*^-^*crnB*^-^) and *nirA* deletion mutant (*nirA*Δ) were used in the growth experiment. Flavohaemoglobin and transporter mutants were also combined with a *niaD*Δ mutation. After 72 h at 37°C, growth was documented by light scanning and the figure was composed using Photoshop 8.0.

Growth tests performed under a variety of conditions (Fig. 5) provided support for the important function of the inducible *fhbA* flavohaemoglobin in protecting *A. nidulans* cells from nitrosative damage. The stronger toxic effect of NO in *fhbA*Δ strains could indicate that not only NR, as shown above by enzymatic assays, but also NiR activity is sensitive to NO and that FhbA protects against this damage. Growth tests performed at different ambient pH values (Fig. 8) provided evidence that FhbA function is necessary to allow optimal NiR activity. Whereas the wild-type and *fhbB*Δ strains utilized NO_2_^-^ over the whole range of acidic pH values tested here (pH 6.8 to pH 4.5), the strain carrying *fhbA* deletions (*fhbA*Δ, *fhbA*Δ*fhbB*Δ and *fhbA*Δ*fhbB*Δ*niaD*Δ) showed increasing sensitivity to 10 mM NO_2_^-^ when ambient pH was reduced stepwise. As the growth reduction could also be due to generally higher NO stress susceptibility in *fhbA*Δ strains and only indirectly related to NiR activity we tested growth in the presence of an additional nitrogen source, 3 mM l-arginine. When growth became independent from utilization of NO_2_^-^ as the sole N-source and hence on functional NiR, the *fhbA*Δ strains grew as well as the wild type over the whole range of pH conditions. These data support the results from growth tests using the NO donor DetaNONOate (compare Fig. 6, row NO_2_^-^ + NO) and strongly suggest that NiR activity is sensitive to high levels of NO and that FhbA function is required for NiR activities under these conditions. Unfortunately, because of the significant spontaneous oxidation of NO to NO_2_^-^ we were not able to directly measure NiR activities under these conditions (data not shown). Finally, an interesting phenotype was obtained in these pH dependence tests in strains lacking either NiR (*niiA*^-^) or NirA (*nirA*^-^, *nirA*Δ) activity. In both genetic backgrounds the utilization of arginine was impaired in the presence of NO_2_^-^ already at the standard pH 6.8 of the medium and was almost totally inhibited as pH gradually decreased (Fig. 8, lanes *niiA*^-^, *nirA*^-^ and *nirA*Δ: compare rows arg at different pH with rows NO_2_^-^/arg at different pH). These phenotypes indicate that high intracellular NO_2_^-^ levels (present in *niiA*^-^ and *nirA*^-^ strains incubated on NO_2_^-^ + arginine; T. Schinko and J. Strauss, unpubl. obs.) negatively influence transporters, enzymes or regulators of the arginine catabolic pathway. At the moment it is not clear whether the utilization of other amino acids is also affected by NO_2_^-^ at low ambient pH. As no typical NO synthetase producing NO and citrulline from l-arginine has yet been identified in fungi, it will be interesting in future to see if NO generating and detoxifying activities in *A. nidulans* may be related to arginine metabolism.

## Discussion

We have shown here that the nitrate assimilation process in *A. nidulans* remodels central components of the nitrogen metabolism. As expected from previous studies, genes involved in uptake and reduction of nitrate to ammonium are induced by the main transcriptional activator NirA and all of these pathway-specific genes carry functionally characterized or putative NirA binding sites (consensus 5′CTCCGHGG). Although more than 800 genes in the genome show the presence of NirA consensus sites within 1 kb of their 5′region, only roughly 80 genes are positively regulated by NO_3_^-^. The vast majority of these genes require nitrate metabolism, and thus NirA, for this response, but only 19 of these NO_3_^-^-responsive genes carry confirmed or predicted NirA consensus sites in their promoters.

We identified more than 50 novel genes, which were upregulated by nitrate and some of them encode enzymes that are required for the subsequent metabolization of the assimilation product (NH_4_^+^), e.g. two types of glutamate dehydrogenases, glutamine synthetase, and three enzymes involved in leucine and coenzyme-A biosynthesis. Notably, the latter genes and some additional genes coding for amino acid transport and metabolism also responded to N-limiting conditions (60 min starvation). This regulatory response suggests that under our conditions (30 min NO_3_^-^ induction after 30 min of N-starvation) the amount of NO_3_^-^ channelled through the assimilatory system was still not producing sufficient amounts of assimilation products as to fully relieve the starvation response. This metabolic status was also seen in direct amino acid measurements. Despite induced NR activity on NO_3_^-^ medium (Fig. S1), the intracellular level of glutamine, which is the molecule known to signal the intracellular nitrogen status(Margelis *et al*., 2001; Berger *et al*., 2008), continued to decline during the first 30 min of nitrate assimilation, identical to the falling Gln levels under N-starvation (Table S1). This apparent discrepancy could be explained by the increasing metabolic demand for amino acids in NO_3_^-^-induced cells, which presumably resumed growth after NO_3_^-^ supply. It was only after approximately 60 min of continuous assimilation that the pool of free Gln started to increase again reaching a steady-state level of balanced repletion/depletion after roughly 2 h. Because little is known about the eukaryotic nitrate signalling cascade, we were mainly interested in the short-term response to nitrate. Thus, our experimental set-up was generally designed to largely avoid the interference with nitrate metabolism, using short incubation times as well as a *nirA*^-^ mutant unable to turn on the assimilatory system. Doing so, we identified several putative regulatory components that might be involved in the signalling cascade, e.g. hypothetical proteins with similarity to guanylate kinases (AN10188), protein kinases (AN3181), transcription factors (AN7343, AN5405) or GTP-binding proteins (AN7222). On the other hand, several genes were repressed by the addition of NO_3_^-^ (predicted transcription factor AN10059, kinase AN10082 or nucleotide-binding domain protein AN10344) and these putative regulators might also be involved in NO_3_^-^-specific signalling.

A novel target of NirA was found to be a flavohaemoglobin-encoding gene (AN7169). This is a member of a gene family encoding evolutionarily conserved proteins, which appear to be widely distributed in bacteria and fungi. Their main role is to metabolize NO radicals, reactive nitrogen species implicated in protein nitrosylation, signalling and nitrosative stress. Flavohaemoglobins are known to catalyse NO di-oxgenation, the NADPH, FAD and O_2_-dependent conversion of NO to NO_3_^-^ (Gardner *et al*., 1998; Poole and Hughes, 2000). In fungi they have been studied in several systems. *Saccharomyces cerevisiae yhb*1Δ (Liu *et al*., 2000), *Cr. neoformans fhb*1Δ (de Jesus-Berrios *et al*., 2003) or *Candida albicans yhb*1Δ (Hromatka *et al*., 2005) lack both significant aerobic NO metabolic activity and detoxification, and therefore are more sensitive to toxic effects of NO. Flavohaemoglobins also work as defence system during the host response to microbial pathogens and mutants show reduced virulence (Crawford and Goldberg, 1998; Idnurm *et al*., 2004). NO detoxification systems might be of significant importance to different fungal species in their natural environments. For pathogenic fungi such as *Aspergillus fumigatus*, detoxification of NO produced as defence molecule by macrophages during the infection process could be essential to counteract nitrosative stress and enhance the infection process. Such a defence system has already been observed for *Cr. neoformans* to be one of the pathogenicity factors (de Jesus-Berrios *et al*., 2003) and *Histoplasma capsulatum*, a pathogenic fungus not known to possess flavohaemoglobins, which uses a nitric oxide reductase to counteract the NO burst employed by infected macrophages as part of the antimicrobial defence system (Nittler *et al*., 2005). *A. nidulans* is not pathogenic but grows on a wide variety of organic substrates and is a common soil inhabitant. In such environments elevated NO concentrations can be released from NO_2_^-^ at acidic pH and it is known that a side-reaction of bacterial nitrification results in release of NO (Klotz and Stein, 2008). This reaction product or derivatives thereof such as nitrous oxide or peroxynitrite may impose nitrosative stress on the surrounding microbial community.

In aspergilli, flavohaemoglobins have been biochemically characterized as NO dioxygenases (Zhou *et al*., 2009) with an additional, and unexpected function in promoting oxidative stress (Zhou *et al*., 2010). Phylogenetic analysis of fungal flavohaemoglobin proteins showed the presence of an *Aspergillus* sp.-specific group, which was proposed to have a function in mycelial branching (Te Biesebeke *et al*., 2010).

While the enzymatic activity of filamentous fungal Fhb enzymes has been confirmed *in vitro* (Zhou *et al*., 2009), their regulatory context and *in vivo* role has not yet been elucidated. Our characterization of the two flavohaemoglobins during nitrate induction yielded novel insights not only into nitric oxide metabolism, but also nitrate and nitrite metabolism in *A. nidulans*. As expected, deletion of the NO-metabolizing genes resulted in higher susceptibility to nitrosative stress imposed by externally supplied nitric oxide. Enzymatic measurements and growth tests, however, provided solid evidence that both NR and NiR are protected by the inducible FhbA flavohaemoglobin from damage by high NO concentrations. As both NR and NiR carry Fe^++^-containing prosthetic groups (haem and Fe-S cluster respectively) this protective function of Fhb proteins might be relevant to nitrogen metabolism under special ecological conditions, such as high NO_2_^-^ concentrations at low external pH, which is known to spontaneously generate high NO concentrations. In plants, NO has been shown to act as a signalling molecule for growth, development and resistance to biotic and abiotic stress (Perazzolli *et al*., 2006). Although the synthesis of NO in plants by enzymatic activities of nitric oxide synthases still remains a matter of debate (Gas *et al*., 2009), intracellular NO generation from NO_2_^-^ by NR activity and spontaneous decomposition at low pH is well established (reviewed by Yamasaki, 2000). Plant flavohaemoglobins, termed non-symbiotic haemoglobins, have been shown to be induced by NO_3_^-^ in *A. thaliana* (Wang *et al*., 2000) and were shown to be responsive to NO_3_^-^, NO_2_^-^ and NO donors in cultured rice cells (Ohwaki *et al*., 2005). Interestingly, also in rice cells, the expression of flavohaemoglobin GLB1 genes is not repressible by nitrogen metabolites (Gln or Asn), identical to what we found for *A. nidulans fhbA*. Recently, NO has also been shown to mediate a negative function on the expression of several nitrate-responsive genes in *Chlamydomonas* and *Arabidopsis* (de Montaigu *et al*., 2010). Low concentrations of NO (10 µ; DetaNONOate) efficiently repressed NO_3_^-^-induced genes via a cGMP-mediated pathway, effectively mimicking nitrogen metabolite repression by ammonium. Whether or not low concentrations of DetaNONOate mediate a negative effect on nitrate assimilatory gene expression in *A. nidulans* is not yet known.

**Fig. 9:**
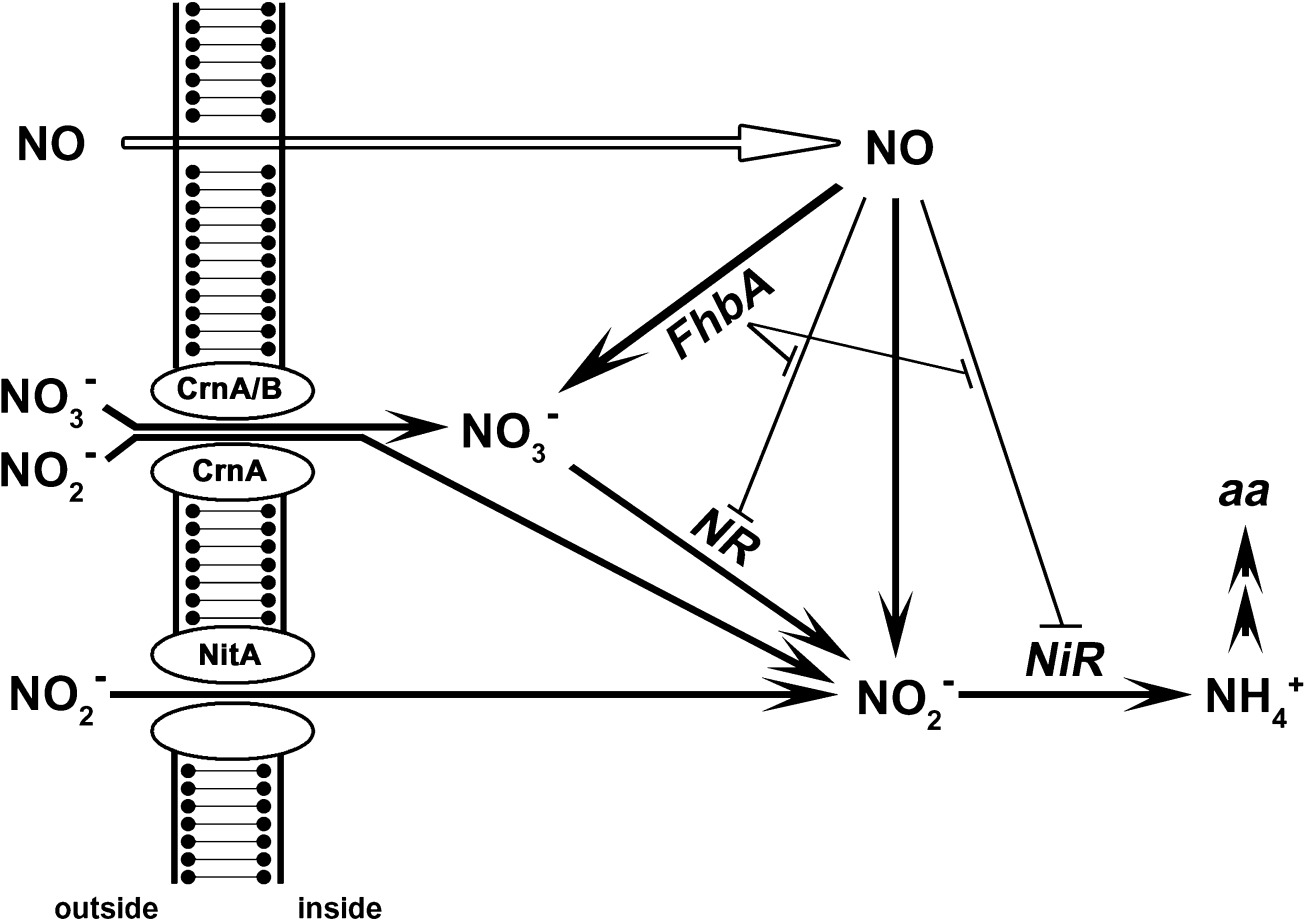
Schematic overview of the interaction between the nitrate assimilation and NO detoxification pathways in *A. nidulans*. Uptake of nitrate (NO_3_^-^) and nitrite (NO_2_^-^) from the outside is enabled by transporters localized in the plasma membrane whereas nitric oxide (NO) passes the plasma membrane by free diffusion independent of any transport system. Following uptake NO_3_^-^ is reduced to NO_2_^-^ by nitrate reductase (NR) and nitrite reductase (NiR) further reduces NO_2_^-^ to NH_4_^+^. Ammonium is incorporated into the cellular amino acid pool (aa) via glutamate and glutamine biosynthesis. Reactive nitric oxide diffuses into the cell and is converted to NO_3_^-^ mainly via the function of the inducible flavohaemoglobins (FhbA). Additionally, NO spontaneously oxidises to NO_2_^-^. This conversion is reversible at a low prevailing pH, e.g. in the fungal vacuole. NO_3_^-^ and NO_2_^-^, originating from NO, again serves as substrate for NR or NiR. Results from enzyme assays and growth tests suggest that elevated intracellular NO levels reduce the function of both NR and NiR and thus FhbA seems to possess a protective function for these two central enzymes in the nitrate assimilation pathway.

*FhbA* is the first reported AreA-independent NirA target thus escaping nitrogen metabolite repression. This regulation could reflect the important role of counteracting NO stress, regardless of the cellular nitrogen status. Using NirA as one of the principal transcriptional activators of FhbA seems to be a good choice as the presence of NO inside or outside the cell always results in spontaneous NO_2_^-^ generation and thus activation of NirA. In addition, intracellular NO may be converted to NO_3_^-^ by the constitutive FhbB protein also leading to NirA activation and subsequent upregulation of FhbA. This regulatory circuit, presented in Fig. 9, ensures an optimal counterstrike against internal and external NO stress and provides protection for the by-products of their enzymatic reactions. Furthermore, the use of membrane-permeable NO has provided strong evidence that in *A. nidulans*, the nitrate transporters do not seem to be involved in transmitting the induction signal to the pathway-specific activator NirA and the intracellular presence of NO, NO_3_^-^ or NO_2_^-^ is evidently sufficient to activate NirA. These results agree with earlier findings by Unkles *et al*. (2001) who reported induction of *niiA* and *niaD* by external NO_3_^-^ in *nrtA/crnA*, *nrtB/crnB* double mutant cells. Although very young mycelium (germling state) was used in the experiments by Unkles and colleges and nitrate uptake kinetics are different between germlings and the mature mycelium (14 h growth) used in our experiments, transporter-independent sensing for the NirA-mediated induction process remains a distinct possibility. This is in contrast to findings in *Arabidopsis* (Ho *et al*., 2009) and *Chlamydomonas* (Rexach *et al*., 2002) where nitrate transporters have been shown to participate in the signal transduction process.

## Experimental procedures

### Strains, growth conditions and genetic techniques

Genotypes of strains used in this study are listed in Table S7. Combinations of traits were obtained by sexual crosses and strains were grown under standard conditions on liquid or solid glucose minimal medium (GMM) with the appropriate supplements and pH adjusted to 6.8 as previously described (Pontecorvo *et al*., 1953). Final concentrations of nitrogen sources were as following: 5 mM di-ammonium tartrate (Fluka), 10 mM NaNO_3_ (Roth), 10 mM NaNO_2_ (Riedel-de Haen), 3 mM (l)-arginine (Sigma), 0.75–3 mM DetaNONOate (Sigma). For growth experiments on solid GMM at low ambient pH (4.5; 5.0 and 5.5) the pH was adjusted prior to sterilization and agar concentration was increased to 3%. Plates were incubated at 37°C up to 72 h and scanned for documentation (300 dpi). Images were processed by using the Photoshop 8.0 software. Conditions for liquid cultures were generally as previously described (Narendja *et al*., 2002), a detailed description is provided in Supporting Information on *Experimental procedures*. Transcriptional analysis for selected genes (Northern analysis) followed our published procedures (Narendja *et al*., 2002), details for probes and isolation can be found in Supporting Materials. Analysis of intracellular nitrate and nitrite levels was performed as previously described for *A. nidulans* by our laboratory (Berger *et al*., 2008) with minor modifications according to Inselsbacher *et al*. (2009). Details are provided in Supporting Information on *Experimental procedures*.

### Microarray experiment

*Aspergillus nidulans* strains used in the transcriptome study were *pabaA*1 (wild type) and the *nirA* loss-of-function strain *nirA*637 (Muro-Pastor *et al*., 1999) *pabaA*1 (*nirA*^-^). A full description of the experimental set-up is found in Supporting Materials and Methods. Briefly, strains were initially pre-grown on GMM for 16 h with 5 mM di-ammonium-tartrate as nitrogen source and then switched to either ammonium (repressed conditions) or to N-free medium for 30 min to prepare the cells for nitrate induction (induced conditions) or for continuous nitrogen depletion (−N conditions). For the wild-type experiments comparing repressed versus induced conditions, seven biologically independent experiments were performed and analysed separately. For the experiments comparing wild type repressed versus N-starved and *nirA*^-^ comparing repressed versus induced, four independent experiments were each performed and analysed separately.

#### Analysis of microarray data

The experimental dataset with details on the microarray obtained from TIGR-PFGRC (The Pathogen Functional Genomics Resource Center; http://pfgrc.jcvi.org/index.php/microarray/protocols.html) has been submitted to the GEO database with accession number GSE10475. For the three conditions, further data analysis was performed in the Biconductor R package (http://www.bioconductor.org/) using the same criteria. Raw median intensity values were imported into R for statistical analysis using the Limma package. Data were normalized within each array using Loess normalization and between arrays using the Aquantile normalization algorithm (Smyth and Speed, 2003). The duplicate correlation function was used to calculate replicate correlations (Smyth *et al*., 2005). The log_2_ expression values for each probe were fitted to a linear model using lmFit (Linear Model for Series of Arrays) and moderated *t*-statistics were calculated by eBayes (empirical Bayes Statistics for Differential Expression) (Smyth, 2004). Genes were considered to be differentially expressed by each treatment if they satisfied both the *P*-value lower than 0.05 and at least a fourfold change.

### Functional enrichment in DEGs

Proteins are functionally classified using the MIPS Functional catalogue (FunCat) (Ruepp *et al*., 2004). Genome-wide data were retrieved from the Pedant *A. nidulans* database (http://pedant.gsf.de/pedant3htmlview/pedant3view?Method=analysis&Db=p3_p130_Asp_nidul) (Walter *et al*., 2009). To statistically assess functional enrichment of DEGs, the probability was calculated using the hypergeometric distribution. In the equation, *m* is the number of genes that contain the same FunCat in *n* selected DEGs, relative to *M* genes that contain the FunCat in all *N* genes of a genome. The functional enrichment of each FunCat was considered significant if the *P*-value was < 0.05.


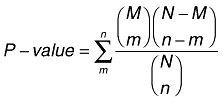


NirA binding sites were identified in the genome using the ‘*fuzznuc*’ program from the EMBOSS package (Rice *et al*., 2000) for regular expression search in nucleotide sequences. The regular expression of the NirA binding site was defined as 5′-CTCCG[ATC]GG and both strands were searched.

#### Construction of *fhbA* and *fhbB* deletion cassettes

The two putative flavohaemoglobin genes designated flavohaemoglobin A (*fhbA*, AN7169.3) and flavohaemoglobin B (*fhbB*, AN3522.3), were deleted using overlapping chimeric polymerase chain reaction fragments produced according to published procedures (Yu *et al*., 2004) and described in detail in Supporting Information on *Experimental procedures*. The primers employed for fragment generation are described in Table S7.

#### Nitric oxide (NO) quantification [(DETC)_2_-Fe2^*+*^–NO; EPR]

Quantification of NO was performed according to Xu *et al*. (Xu *et al*., 2005). In brief, approximately 0.3 g of frozen sample powder was mixed with 800 µl phosphate buffer (100 mM) containing 0.32 M sucrose, 0.1 mM EDTA and 5 mM thioethylenglycol (pH 7.4). After centrifugation (13 000 *g*, 20 min, 4°C) the supernatant was incubated with 400 µl of the spin trapping reagent for 60 min. The stock solutions for the spin trap reagent consisted of 7.5 mM aqueous FeSO_4_ and 15 mM aqueous DETC containing 0.5 M Na_2_S_2_O_4_. FeSO_4_ and DETC solutions were mixed 1:1 just before addition to the sample solution. Ethyl acetate (800 µl) was subsequently added and the mixture was then shaken for 3 min. After centrifugation (13 000 *g*, 6 min, 4°C), the organic solvent layer was transferred into a quartz flat cell for EPR measurement.

Electron paramagnetic resonance spectra were acquired as first derivatives of the microwave absorption with a Bruker EMX CW spectrometer, operating at X-band frequencies (9 GHz) and using a high sensitivity cavity. Microwaves were generated by a Gunn diode and the microwave frequency was recorded continuously with an in-line frequency counter. Spectra were recorded using 20 mW microwave power, 100 kHz modulation frequency and 1 G modulation amplitude. Signal intensities were determined by double integration using the Bruker WINEPR software, and then were corrected for the dry weight of input material.

### NR activity assay

Nitrate reductase activity measurements are based on formation of nitrite from externally added nitrate in cellular extracts and nitrite formation was quantified with the diazo-dye adduct colorimetric kit (Roche) at 540 nm. Details of the experimental set-up are given in Supporting Information on *Experimental procedures*.
