# Supplementary material for: Transcriptome analysis of nitrate assimilation in Aspergillus nidulans reveals connections to nitric oxide metabolism
Source: Mol Microbiol. 2010 Sep 27;78(3):720–38. doi: 10.1111/j.1365-2958.2010.07363.x (PMC3020322; doi:10.1111/j.1365-2958.2010.07363.x)
Supplement: Supplementary file 1 [file mmi0078-0720-SD1.pdf]

Supporting Information on Experimental Procedures and Materials

Growth conditions for different nitrogen treatments and genetic techniques

A. nidulans strains were grown for 14 h at 37°C in a rotary shaker (180 rpm). (L)-arginine (3 mM) served as nitrogen source for pre-growth, except for the microarray experiment in which ammonium was sued as N-source. After pre-cultivation biomass was collected by filtration through Miracloth (Calbiochem), washed with sterile GMM, and resuspended in fresh GMM (120 ml) lacking any nitrogen source. Aliquots (30 ml) were further incubated for 30 minutes in this medium and subsequently different nitrogen sources were added to specify the condition. Repressed [NH_4], 5 mM di-ammonium tartrate; repressed-induced NO [NH_4^{NO}], 5 mM di-ammonium (D)-tartrate + 1.5 mM DetaNONOate; non induced [NI], 3 mM (L)-arginine; induced NO_3^- [NO_3], 3 mM (L)-arginine + 10 mM $NaNO_3$; induced nitric oxide (Adams *et al.*): 3 mM (L)-arginine + 1.5 mM DetaNONOate). Biomass was harvested by filtration, washed with cold deionised H_2O and biomass was split into portions for subsequent parallel analysis of different markers (nitrate reductase activity assay, transcriptional analysis, and measurement of intracellular nitrate/ nitrite or nitric oxide). Transformation of *A. nidulans* strains was performed as described (Tilburn et al., 1983) using 500ng of PCR fragment. Correct integration events for the *fhbA* and *fhbB* deletion PCR fragments were verified by Southern and PCR analysis using primers flav 35 P F check / flav35 T R check or flav 71 P F check / flav 71 T R check, respectively (primer sequences are given in Supporting Table ST 8).

Growth conditions for the generation of RNA for transcriptome analysis

After pregrowth on GMM for 16 hours with 5mM di-ammonium-tartrate as nitrogen source cells were harvested, washed and replaced in fresh minimal media either lacking any nitrogen source (for induced and starved conditions) or containing 10mM ammonium (for repressed

conditions). After 30 minutes of incubation in N-free medium, 10mM $NaNO_3$ (in 2ml of 1M $NaNO_3$) was added to generate inducing conditions, or 2 ml sterile water was added to generate N-starved conditions. Both types of samples were further incubated for 30 minutes before harvesting for RNA extraction. For generating repressed conditions, cells incubated on 10 mM NH_4^+ were further incubated for 30 minutes on this nitrogen source and finally harvested for RNA extraction. Mycelia were harvested by filtration and quickly frozen in liquid nitrogen. For total RNA isolation, the mycelia were disrupted by grinding in liquid nitrogen and total RNA was extracted with Trizol reagent (Invitrogen). Ten micrograms of RNA from each treatment were then fractionated in a 0.8% agarose gel, stained with ethidium bromide, and then visualized with UV-light. The presence of intact 28S and 18S ribosomal RNA bands was used as a criterion to assess the integrity of the RNA. For the wild type experiments comparing repressed versus induced conditions seven biologically independent experiments were performed and analyzed separately. For the experiments comparing wild type repressed versus N-starved and *nirA*$^-$ comparing repressed versus induced, four independent experiments were performed each and analyzed separately. From each experiment 10 µg of total RNA samples were labelled by incorporation of aminoallyl-dUTP followed by a coupling of the aminoallyl groups to either Cyanine-3 or Cyanine-5 and hybridized according to the provided protocol (see protocols at http://pfgrc.tigr.org/protocols.shtml). For scanning, an Agilent DNA Microarray scanner G 25 65 B was used to obtain TIFF files. These were processed using Agilent Feature extractor software version 9.1.

Determination of intracellular amino acid pools

For determination of intracellular free amino acid pools, wild type cells were incubated in triplicate under the conditions described above. Samples were harvested after 16h pre-growth (time point -30 min.), and after the 30 min. incubation time on N-free medium (time point 0

min.). For the induction experiment, 10mM NaNO3 was added to the medium and samples were harvested after 10, 20, 30, 40, 50, 60, 90, 120 and 180 minutes of incubation on the inducing medium. For the starvation experiment, samples were harvested directly after pre-growth on ammonium (time point -30 min.), after the initial incubation on N-free medium (time point 0 min.) and subsequently after 30, 60, 90 and 120 minutes of incubation in N-free medium. Treatment of cells for determination of intracellular free amino acid pools and subsequent analysis was performed as described previously (Berger *et al.*, 2008).

Northern Blot analysis

Total RNA was isolated from frozen mycelia using TRIzol ® Reagent (Invitrogen, USA) and previously published protocols were applied for Northern blotting and hybridization(Narendja *et al.*, 2002). Membranes were hybridized with ^{32}P (PerkinElmer) labelled probes derived from PCR products of *niaD*, *niiA*, *fhbA*, *acnA* and 18*S* genes (see Supporting Table ST 8 for primer sequences). Hybridization signals were analyzed on a phosphoimager (Storm; Molecular Dynamics, Inc.) and signal intensities were quantified using the Image-Quant (Molecular Dynamics) software. Specific signals were normalized to actin (*acnA*) or 18*S* values used as loading controls. Calculations included at least two independent biological experiments.

Construction of *fhbA and fhbB* deletion cassettes

The two putative flavohemoglobin genes designated flavohemoglobin A (*fhbA*, AN7169.3), and flavohemoglobin B (*fhbB*, AN3522.3), were deleted using overlapping chimeric PCR fragments constructed according to published procedures (Yu *et al.*, 2004). Primer sequences used for construction of the deletion cassettes are listed in Supporting Table ST 8. For cloning the arginine auxotrophy marker *argB* (AN04409) sequence obtained from pMS12 (http://www.fgsc.net/plasmid/nidplas.html), harbouring a *ClaI* restriction site at the 5'-end or

a *BamHI* restriction site at the 3'-end, was used. The upstream region of AN3522.3 was amplified with primers flav 35 Pr F and flav 35 Pr R ClaI using REDTaq® ReadyMix™ (Sigma Aldrich) from genomic wild type DNA and the resulting fragment (~ 3.9 kb) was ligated to *argB* via generated *ClaI* site. The downstream fragment of the AN3522.3 was amplified using primers flav 35 T F BamHI and flav 35 T R ligated to *argB* via introduced *BamHI* restriction site. The chimeric ligation products fragments were used for High Fidelity PCR Mix (Fermentas) with primers flav 35 Pr F / arg flavo ko R and arg flavo ko F / flav 35 T R, respectively. 1 µg of each PCR product was used for transformation. The construction of the AN7169.3 deletion cassette followed the same scheme. AN7169.3 upstream region was obtained by PCR using primers flav 71 Pr F and flav 71 Pr R EcoRI and ligated to an *argB* fragment bearing a *EcoRI* restriction site at the 5'-end. The downstream sequence of AN7169.3 was obtained using primers flav 71 T F ClaI and flav 71 T R and ligation to *argB* coding sequence possessing a *ClaI* at the 3'-end. The chimeric fragments were amplified using High Fidelity PCR Mix (Fermentas) with primers combination flav 71 pr F / arg flavo ko R or arg flavo ko F / flav 71 T R, and 1 µg of PCR product was used for transformation.

Construction of *nirA* deletion cassettes

A complete *nirA* (AN0098) deletion mutant was generated by replacement of *nirA* open reading frame (ORF) by $riboB_{fum}$ (Afu1*g*13300) coding sequence complementing riboflavin auxotrophy. The upstream region of *AN*0098 was amplified with primers nirA Pr-1790 F and nirA Pr-1 ClaI R using REDTaq® ReadyMix™ (Sigma Aldrich) and genomic wild type DNA. Likewise the downstream region was amplified with primers nirA T+1 BamHI F and nirA T+1658 R. Together with the $riboB_{fum}$ fragment, possessing a *ClaI* and *BamHI* restriction site, the promoter (~ 1.7 kb) and terminator (~ 1.7 kb) fragments were ligated into pGEM-Teasy plasmid (Promega) obtaining the *nirA* deletion cassette. For transformation the cassette was

NotI released from the plasmid and 1 µg of the linear fragment used for transformation. Deletion of *nirA* was screened by PCR using primers nirA Pr-1790 F / nirA 557 R and confirmed by Southern blot analysis.

Construction of *niaD* deletion cassettes

To generate a nitrate reductase (*niaD*) mutant in a nitrate transporter deficient *A. nidulans* strain the AN1006 ORF was disrupted and partially deleted by the insertion of an *argB* coding sequence derived from pMS12. The 5'-fragment of the *niaD* ORF was amplified by PCR using genomic wild type DNA and primer niaD ATG F and niaD 1428 EcoRI R. Use of primer niaD 1557 BamHI F and niaD 2957 R yielded the 3'-fragment of the niaD ORF. Together with the *argB* coding fragment, possessing *EcoRI* and *BamHI* restriction site, the 5'-ORF (~ 1.4 kb) and 3'-ORF (~ 1.4 kb) fragments were ligated into pGEM-Teasy plasmid (Promega) via T/A overhangs obtaining the *niaD* deletion cassette. For transformation the cassette was *NotI* released from the plasmid and 1 µg of the linear fragment used for transformation of strain *crnA*$^{-}$ *crnB*$^{-}$. *niaD* replacement was screened by PCR using primer niaD F / arg flavo ko R and niaD ATG F / niaD 2957 R and confirmed by Southern blot analysis.

Intracellular NO_3^- and NO_2^- measurements

The method is based on published procedures by Narendja et al., 2002, Miranda et al., 2001 and Inselbacher et al., 2009 with minor modifications as described herein. Approximately 150 mg frozen mycelia (wet weight) were extracted with 1 ml 70% ethanol, using 200 µl glass beads (0,75-1 mm, Roth) in a RiboLyser (Hybaid, amplitude 4.5, 45 seconds). After pelleting of cell debris (Biofuge pico, Heraeus, 13,000rpm, 4°C) the supernatant was transferred into a fresh 2 ml Eppendorf tube, and the pellet re-extraced once again. Pooled phases were evaporated in a Speed Vac Concentrator (Savant). Until further analysis samples were stored

at -20 °C. Dried sample were dissolved in 600 µl bi-destilled water and turbidity reduced by extraction with equal volume of chloroform. Further measurement was performed in 96 wells microtitre plates. 50 µl of the aqueous layer were mixed with 50 µl vanadium(III)chloride solution (Sigma, 0.8% (w/v) in 1 M HCl) chemically reducing present NO_3^- to NO_2^-. Subsequently, 50 µl of a 1:1 mix of N-(1-Naphthyl)ethylendiamine didydrochloride (Sigma, NEDD, 0.02% (w/v)) and sulfanilamide (Sigma, SULF, 1% (w/v)) were added and the plate incubated at 37 °C for 45 minutes. Absorbance of the coloured product was measured at 540 nm using a plate reader (Victor2, Wallac, 1420 Multilabel Counter) representing total NO_2^- levels. To quantify NO_2^- concentrations addition of vanadium(III)chloride was skipped. Nitrogen standards, NO_3^- or NO_2^-, ranging from 0.02 to 5 ppm nitrogen were used for calculation of NO_3^- and NO_2^- values. NO_3^- was calculated as difference of total NO_2^-, including chemically reduced NO_3^-, and intrinsic NO_2^- amounts. Detected µg of NO_3^- or NO_2^- were normalized to dry weight (DW) values of the extracted biomass, after 72 h at 60°C, resulting in µg NO_3^- or NO_2^- g-1 DW ratios. For detection of low NO_3^- values in presence of high NO_2^- background the analytical setup was limited. Analysis of NO_3^- standards in presence of increasing NO_2^- showed bias from standard dilutions without NO_2^- addition. Therefore, NO_3^- levels of WT [NO], *fhbA*Δ *fhbB*Δ [NO] and *nirA*⁻ [NO] samples, showing high NO_2^- concentrations, are regarded as non detectable (n.d.). Results of a representative experiment, including standard deviation of a technical repetition are given in Figure 6.

Nitrate reductase (NR) activity assay.

After cultivation on various N-sources, 200 mg mycelia (wet weight) were directly processed without an intermediate freezing step. Aliquots were placed in 1.5 ml reaction tubes containing 500 µl glass beads (0.75-1 mm) and 500 µl pre-chilled protein extraction buffer (100 mM phosphate buffer pH 7.5, 1 mM EDTA, 1 mM PMSF and a protease inhibitors (Sigma). Cells were broken using the Ribolyzer (Hybaid) at settings amplitude 4.5 for 35

seconds. Prior and after cell disruption samples were cooled on ice. Cell debris was sedimented by centrifugation (4°C, 13,000 rpm, 15 min), the supernatant was recovered and stored on ice until further use. The protein concentration of cell lysates was determined using BCA assay (Pierce) and samples were diluted to a protein concentration of 1 mg/ml. The nitrate reductase assay mixture comprised 100 µl 8 mM $NaNO_3$, 50 µl NAD(P)H/FAD solution, prepared according to manufacturer's instructions (Roche), and 4 µl protein extract (1 mg/ml) or 4 µl fungal nitrate reductase (0,005 – 1 U/ml) supplied with the kit. Mixtures were incubated at 25°C for 30 minutes before addition of 50 µl colour reagent I and 50 µl colour reagent II. The reaction of NO_2^- with sulfanilamide and N-(1-naphthyl)-ethylene-diamine resulting in the diazo-dye adduct was detected (540 nm) after 15 minutes of incubation in the dark. Units were calculated by referring to the linear range of the nitrate reductase calibration curve obtained with purified NR from the kit.

Supplementary Table 1

Amino acid levels (nmol/mg DW) in the wild type at time point 0 (after starvation period of 30 min.) and subsequently after induction by 10 mM NO3

time[min]	0		10		20		30		40		50		60		90		120		180	
	mean	sd	mean	sd	mean	sd	mean	sd	mean	sd	mean	sd	mean	sd	mean	sd	mean	sd	mean	sd
Asp	11,45	2,34	14,54	3,71	14,86	4,87	14,66	3,34	12,46	4,01	11,16	2,94	8,52	0,44	8,37	0,99	9,39	1,06	12,05	1,74
Glu	61,77	1,71	68,53	1,46	68,31	3,72	69,05	1,46	60,66	0,86	54,75	2,43	49,96	5,42	54,64	6,13	57,55	0,63	63,04	8,72
Asn	14,49	1,48	14,41	0,10	10,67	0,76	7,07	1,01	4,11	0,79	3,04	1,33	2,69	0,95	3,95	1,06	4,58	0,68	6,28	0,853
Ser	7,62	0,95	8,25	0,15	8,06	0,49	7,33	1,29	6,72	0,56	7,07	0,42	7,21	0,68	7,07	1,19	6,70	0,09	7,63	1,146
His	4,08	0,47	4,40	0,62	4,20	0,17	4,50	0,04	5,09	1,62	5,40	1,35	4,85	1,31	2,90	0,64	2,44	0,64	2,29	0,771
Gln	61,82	10,56	58,69	5,24	47,75	4,44	34,98	9,80	20,99	9,64	16,78	9,23	22,78	6,39	60,82	2,37	49,82	3,58	70,07	2,754
Gly	7,32	2,36	7,35	0,38	6,82	1,43	5,03	1,97	3,63	1,17	2,94	1,13	2,72	1,08	2,31	1,20	1,83	0,77	2,39	1,208
Thr	3,18	0,02	3,14	0,01	3,05	0,02	2,78	0,18	2,65	0,44	2,53	0,38	2,32	0,37	2,16	0,21	2,10	0,03	2,54	0,226
Arg	32,08	3,54	33,88	3,07	33,47	2,03	34,49	4,14	30,20	2,91	30,63	1,04	29,83	1,42	26,79	4,21	23,92	1,53	23,58	3,166
Ala	12,33	5,91	13,58	2,92	14,90	4,02	11,95	4,57	9,53	4,88	7,51	5,05	7,20	4,78	12,00	5,51	10,57	2,31	16,73	4,173
Tyr	1,07	0,46	0,92	0,37	0,84	0,36	0,80	0,40	0,76	0,37	0,77	0,31	0,74	0,28	0,82	0,43	0,74	0,43	0,82	0,404
Val	2,58	0,63	2,28	0,76	1,89	0,64	1,69	0,60	1,66	0,60	1,62	0,63	1,55	0,63	1,76	0,89	1,49	0,61	1,76	0,751
Ile	0,89	0,12	0,76	0,18	0,63	0,15	0,55	0,14	0,54	0,14	0,54	0,17	0,51	0,18	0,53	0,30	0,45	0,23	0,58	0,297
Phe	0,25	0,10	0,18	0,06	0,15	0,04	0,14	0,03	0,15	0,04	0,21	0,05	0,22	0,03	0,24	0,10	0,20	0,09	0,26	0,133
Leu	1,00	0,30	0,82	0,25	0,87	0,19	0,86	0,16	0,96	0,20	1,07	0,13	1,07	0,17	0,98	0,47	0,87	0,37	1,04	0,431
Lys	16,52	3,45	17,11	2,78	17,17	3,77	17,26	4,07	15,10	3,97	14,51	1,69	6,17	0,86	11,39	5,04	9,98	4,66	9,53	4,616
Met	0,48	0,12	0,44	0,05	0,44	0,00	0,40	0,05	0,37	0,00	0,40	0,01	0,39	0,01	0,44	0,05	0,42	0,04	0,52	0,05

Amino acid levels (nmol/mg DW)in the wild type at the beginning of starvation period (-30), at time point 0 (after starvation period of 30 min.) and subsequent incubation in the absence of any nitrogen source

time[min]	-30		0		30		60		90		120	
	mean	sd	mean	sd	mean	sd	mean	sd	mean	sd	mean	sd
Asp	13,21	1,33	7,507	0,179	20,27	0,369	22,99	0,777	19,6	1,734	17,67	1,363
Glu	65,17	5,05	44,14	0,945	74,4	1,12	66,94	1,753	48,76	2,439	54,65	3,543
Asn	18,72	1,95	20,05	0,662	18,93	0,183	10,66	0,397	1,98	0,24	1,7	0,298
Ser	8,632	0,45	6,387	0,395	9,138	0,159	6,946	0,168	5,972	0,007	6,912	0,753
His	6,895	0,56	5,52	0,268	5,959	0,167	6,177	0,234	6,717	1,046	6,414	0,716
Gln	148,3	9,14	94,24	2,291	61,9	0,861	32,36	0,953	7,218	0,305	9,254	0,737
Gly	7,132	0,29	5,43	0,859	6,596	0,103	4,118	0,127	2,604	0,069	3,183	0,645
Thr	3,196	0,2	3,329	0,097	3,371	0,04	2,744	0,191	2,904	0,294	3,162	0,428
Arg	41,19	2,81	35,01	0,921	39,67	0,458	36,54	1,038	33,3	0,99	31,25	2,871
Ala	37,62	1,23	23,67	0,292	20,89	0,507	13,98	0,047	5,761	0,022	8,546	1,026
Tyr	1,392	0,05	1,911	0,045	1,455	0,131	1,198	0,027	1,368	0,026	1,263	0,084
Val	2,44	0,2	4,147	0,114	2,513	0,022	1,537	0,02	1,492	0,102	1,516	0,259
Ile	0,911	0,06	1,756	0,072	0,908	0,006	0,517	0,007	0,781	0,015	0,72	0,081
Phe	0	0	0	0	0	0	0,132	0,015	0,314	0,16	0,497	0,102
Leu	0,895	0,04	1,766	0,032	0,957	0,02	0,918	0,011	1,452	0,052	1,32	0,208
Lys	16,61	0,67	15,25	0,293	18,48	0,463	16,76	0,523	15,37	0,573	15,94	1,851
Met	0,412	0	0,316	0,014	0,273	0,043	0,341	0,018	0,376	0,023	0,367	0,077

Supporting Table ST 1. Levels of free amino acids determined by HPLC according to previously published procedures (Berger et al., 2008). The mean of three independent experiments are shown along with the calculated standard deviation (sd).

Supporting Table ST 2A: Functional Categories (FunCat)

FunCat	6(-d-)	45(d--)	3(--u)	4(-u-)	72(u--)	1(u-u)	1(uu-)	3(uuu)
amino acid metabolism (01.01)		1	1		8	1	1	1
nitrogen, sulfur and selenium metabolism (01.02)	1				5	1	1	1
nucleotide/nucleoside/nucleobase metabolism (01.03)					2			
phosphate metabolism (01.04)					1			
C-compound and carbohydrate metabolism (01.05)	1	2			7			
lipid, fatty acid and isoprenoid metabolism (01.06)		2			4			
metabolism of vitamins, cofactors, and prosthetic groups (01.07)					1			
secondary metabolism (01.20)		2			4			
glycolysis and gluconeogenesis (02.01)					2			
anaplerotic reactions (02.09)					1			
tricarboxylic-acid pathway (citrate cycle, Krebs cycle, TCA cycle) (02.10)					2			1
electron transport and membrane-associated energy conservation (02.11)					1			
respiration (02.13)					1			
fermentation (02.16)					1			
energy conversion and regeneration (02.45)					1			
DNA processing (10.01)		1						
cell cycle (10.03)		2			1			
RNA synthesis (11.02)	1				1			
RNA processing (11.04)	1	2						
protein folding and stabilization (14.01)					2			
protein modification (14.07)		1			2			
protein/peptide degradation (14.13)				1	2			
protein binding (16.01)		2						
nucleic acid binding (16.03)		2						
nucleotide/nucleoside/nucleobase binding (16.19)					1			
complex cofactor/cosubstrate/vitamine binding (16.21)					4			1
transported compounds (substrates) (20.01)	2	1		1	13			2
transport facilities (20.03)		1		1	8			1
transport routes (20.09)	1	1		1	5			1
cellular signalling (30.01)		1						
stress response (32.01)		1			6			
disease, virulence and defense (32.05)		1			3			
detoxification (32.07)		1			2			
degradation / modification of foreign (exogenous) compounds (32.10)					1			
homeostasis (34.01)					2			2
cellular sensing and response to external stimulus (34.11)	1				3			
cell wall (42.01)		2			1			1
fungal/microorganismic cell type differentiation (43.01)		1			2			1

Supporting Table ST2A. Number of DEGs asssigned to functional classifications (reduced to the a second level). The total number of genes affected by the three different conditions are given in front of the brackets, symbols within brackets are according to nomenclature used in the VENN diagram (Figure 2). d, down-regulated, u, up-regulated. The sum of the individual FunCat-assigned genes can exceed the total number of DEGs for a given condition because one gene can be assigned to more than category

Supportiung Table ST2B: Significantly over-represented categories

FunCat	6(-d-)	45(d--)	3(--u)	4(-u-)	72(u--)	1(u-u)	1(uu-)	3(uuu)
amino acid metabolism (01.01)					8	1	1	
nitrogen, sulfur and selenium metabolism (01.02)					5	1	1	
anaplerotic reactions (02.09)					1			
tricarboxylic-acid pathway (citrate cycle, Krebs cycle, TCA cycle) (02.10)					2			1
transported compounds (substrates) (20.01)					13			2
transport facilities (20.03)					8			
homeostasis (34.01)								2
cell wall (42.01)								1

Supporting Table ST2B. Number of genes asssigned to functional classifications (reduced to the a second level). Only categories which are significantly overrepresented compared to the genomic distribution are shown.

Supporting Table 3. ***A. nidulans*** **genes that positively respond to NO3 induction in the wild type**

**Gene number**[1]	**ratio**[2]	**P-value**	**known or putative function**[3]	**NirA-BS**[4]
**AN1007**	5,90	2,64E-07	*niiA* ; nitrite reductase	2 [IGR]
**AN0399**	5,31	4,40E-09	*crnB (nrtB)* ; NO3 transporter high affinity	-454
**AN5120**	4,90	1,08E-02	hypothetical protein	-379
AN2134	4,90	1,58E-02	conserved hypothetical protein	
AN7539	4,77	7,27E-10	putative hydrophobin	
AN3206	4,69	1,75E-02	similar to glucose-methanol-choline (GMC) oxidoreductases	
AN7343	4,50	1,93E-02	predicted Zn(II)2Cys6-domain containing transcription factor	
AN11493	4,42	2,02E-02	hypothetical protein (predicted 54 aa)	
AN3968	4,13	2,36E-02	conserved hypothetical protein	
**AN1008**	4,02	1,85E-07	*crnA (nrtA)* ; NO3 transport high affinity	-767
AN11628	3,89	2,71E-02	hypothetical protein (predicted 51 aa)	
**AN1006**	3,85	9,39E-07	*niaD* ; nitrate reductase	2 [IGR]
AN1791	3,85	2,77E-02	conserved hypothetical protein	
AN5405	3,81	2,84E-02	predicted bZIP-containing transcription factor similar to stress-induced XBP1	
AN5558	3,62	4,89E-08	*alp1* ; starvation-induced vacuolar protease	
**AN10464**	3,60	3,22E-02	conserved hypothetical protein	-84
**AN7169**	3,60	7,66E-07	*fhbA* ; NirA-dependent flavohemoglobin	-225
AN0180	3,59	3,17E-03	predicted enoyl-coA hydratase involved in acetyl coA and lipid metabolism	
AN11316	3,53	3,37E-02	hypothetical protein (predicted 55 aa)	
AN3541	3,53	3,38E-02	conserved hypothetical protein	
AN1261	3,51	2,74E-02	conserved hypothetical protein	
AN1659	3,50	2,80E-08	predicted amino acid transporter	
AN2834	3,43	3,59E-02	conserved hypothetical protein with similarity to GDSL-type lipase	
AN1737	3,43	3,61E-02	conserved hypothetical protein with predicted cyt. P450 domain	
AN10289	3,39	3,70E-02	predicted SM-related DMATS-type aromatic prenyltransferases	
AN7988	3,39	3,70E-02	conserved hypothetical protein	
**AN6414**	3,39	3,70E-02	conserved hypothetical protein with predicted cyt. P450 domain	-673

1 Gene number according to Fungal Genome Database at Broad Institute
2 ratio between NH4-repressed and NO3-induced cells expressed as log2 value
3 established gene function or predicted function according to CADRE Initiative annotation
4 NirA binding sites have been studied for the 4 sites present in the niiA-niaD intergenic region (IGR)
position of other binding sites are derived from bioinformatic analysis of -1kb regions

Gene number[1]	ratio[2]	P-value	known or putative function[3]	NirA-BS[4]
AN9242	3,37	1,82E-02	conserved hypothetical protein	
AN11030	3,32	3,87E-02	predicted NADP-coupled glycerol dehydrogenase	
AN6942	3,28	3,97E-02	conserved hypothetical protein	
AN7358	3,20	1,15E-02	predicted dihydroxy-acid dehydratase; biosynthesis of Val, Leu, Iso and coA	
**AN3611**	3,20	4,21E-02	conserved hypothetical protein	-500
AN3340	3,16	4,31E-02	conserved hypothetical protein	
AN11471	3,16	4,32E-02	hypothetical protein (predicted 51 aa)	
**AN10188**	3,16	4,32E-02	conserved hypothetical protein with guanylate kinase domain	-767
AN0587	3,12	4,46E-02	conserved hypothetical protein with similarity to HSP70 chaperone	
AN6410	3,06	4,62E-02	conserved hypothetical protein	
AN2793	3,04	4,82E-09	*leu2B* ; isopropylmalat-dehydrogenase involved in Leu and Glu biosynthesis	
AN9215	3,04	4,72E-02	hypothetical protein	
AN8027	3,03	1,56E-02	conserved hypothetical protein	
**AN10431**	3,02	4,76E-02	conserved hypothetical protein	-844
AN7222	3,02	4,76E-02	conserved hypothetical protein with GTP-binding domain	
AN4367	2,99	4,89E-02	predicted class-III chitin synthetase	
AN9185	2,98	4,91E-02	conserved hypothetical protein	
AN10049	2,76	7,39E-03	scytalone dehydratase involved in monodictyphenone biosynthesis	
AN11046	2,72	1,24E-06	conserved hypothetical protein	
AN5886	2,71	6,25E-06	*luA* ; isopropylmalat isomerase involved in biosynthesis of Leu	
AN8445	2,65	1,09E-05	predicted mainopeptoidase-Y involved in vacuolar protein degradation	
AN6633	2,53	3,03E-02	conserved hypothetical protein	
AN2602	2,53	3,45E-02	conserved hypothetical protein with lipase/esterase domain	
**AN3181**	2,51	7,72E-05	conserved hypothetical protein with protein kinase domain	-918
AN3504	2,50	6,90E-03	conserved hypothetical protein with similarity to alpha-glucosidase	
AN2944	2,46	2,30E-07	*tamA;* transcriptional co-actictivator interacting with AreA	
**AN8647**	2,44	4,39E-05	*nitA;* nitrite transporter	-344
AN6933	2,44	5,86E-03	conserved hypothetical protein with similarity to mitochondrial malic enzyme	

1 Gene number according to Fungal Genome Database at Broad Institute
2 ratio between NH4-repressed and NO3-induced cells expressed as log2 value
3 established gene function or predicted function according to CADRE Initiative annotation
4 NirA binding sites have been studied for the 4 sites present in the niiA-niaD intergenic region (IGR) position of other binding sites are derived from bioinformatic analysis of -1kb regions

**Gene number**[1]	**ratio**[2]	**P-value**	**known or putative function**[3]	**NirA-BS**[4]
AN6118	2,41	1,31E-02	conserved hypothetical protein with similarity to amino acid transporters	
AN9329	2,40	1,68E-02	conserved hypothetical protein	
AN5771	2,34	1,82E-03	conserved hypothetical protein	
AN0660	2,34	4,91E-04	*furA* ; allantoin transporter protein	
AN1061	2,25	7,19E-06	conserved hypothetical protein with similarity to GABA permeases	
**AN0627**	2,25	1,53E-08	conserved hypothetical protein with PI-3-P membrane binding domain	-803
AN0811	2,23	2,23E-02	conserved hypothetical protein with similarity to amino acid transporters	
AN7769	2,23	3,24E-02	conserved hypothetical protein	
AN6076	2,19	2,78E-02	conserved hypothetical protein with SNF2-type helicase domain	
**AN8439**	2,16	4,06E-02	conserved hypothetical protein	-598
**AN0380**	2,14	4,75E-02	conserved hypothetical protein with similarity to prolyl cis-trans isomerases	-644
AN11489	2,14	2,89E-11	hypothetical protein (predicted 23 aa)	
AN9451	2,08	1,22E-08	conserved hypothetical protein	
AN8557	2,06	3,79E-02	conserved hypothetical protein	
AN8558	2,05	4,11E-02	conserved hypothetical protein	
AN0628	2,00	3,33E-04	predicted D-lactate dehydrogenase involved in pyruvate-acetyl-coA metabolism	

Supporting Table 4. *A. nidulans* genes that positively respond to N-limitation in the wild type and *nirA*$^{-}$ strain

Gene number[1]	Group[3]	ratios in different experiments[2]				known or putative function[4]
		ratio e2	P-value	ratio e3	P-value	
	**WT^{-N}**					
**AN8903**	(-u-)	**3,27**	1,07E-02	1,64	2,31E-03	hypothetical protein with similarity to MFS-family peptide transporter
**AN2572**	(-u-)	**2,94**	2,29E-02	0,81	4,03E-04	hypothetical protein with similarity to dipeptidyl-peptidase
**AN1404**	(-u-)	**2,57**	1,66E-02	-2,43	2,61E-01	conserved hypothetical protein with predicted diacylglycerol kinase domain
**AN1927**	(-u-)	**2,39**	1,22E-02	0,58	6,00E-01	predicted Zn(II)2Cys6-domain containing transcription factor
	**WT^{-N} WT^{NO3}**					
**AN4159**	(uu-)	**2,22**	1,16E-02	1,98	3,14E-03	*glnA* ; glutamine synthetase
	**WT^{all}**					
**AN0418**	(uuu)	**3,85**	1,05E-03	**2,88**	3,90E-05	*dur3* ; urea transporter
**AN4376**	(uuu)	**3,18**	3,73E-04	**4,97**	5,33E-09	*gdhA* ; NADP-specific glutamate dehydrogenase
**AN7463**	(uuu)	**2,43**	7,42E-04	**4,50**	2,16E-06	*meaA* ; low affinity ammonium transporter
	**WT/nirA^{NO3}**					
**AN5134**	(u-u)	1,58	1,87E-02	**2,76**	6,10E-06	*gltA* ; NAD-specific glutamate dehydrogenase
	**nirA^{NO3}**					
**AN3991**	(--u)	1,96	4,71E-02	**5,82**	9,77E-03	hypothetical protein with similarity to glucuronyl hydrolases
**AN7183**	(--u)	1,04	2,00E-01	**2,90**	5,34E-03	conserved hypothetical protein
**AN1731**	(--u)	0,31	4,27E-01	**2,27**	2,77E-04	*prnD* ; proline dehydrogenase

1 Gene number according to Fungal Genome Database at Broad Institute
2 ratio between two conditions in each of the experiments expressed as log2 value
3 (-u-), upregulated in WT/ -N (e2); (uu-), upregulated in WT -N (e2) and WT/NO_3 (e1); (uuu), upregulated in (e1), (e2) and (e3)
(u-u), upregulated in WT/-N (e2) and *nirA*$^{-}$/NO_3 (e3); (--u), upregulated in *nirA*$^{-}$/NO_3
4 established gene function or predicted function according to CADRE Initiative annotation

Supporting Table 5. A. nidulans genes that negatively respond to NO_3 induction in the wild type

Gene number[1]	ratio[2]	P-value	known or putative function[3]	
**AN11308**	-4,29	2,16E-02	hypothetical protein	
**AN10470**	-4,00	2,53E-02	predicted DNA-polymerase III delta subunit	
**AN8788**	-4,00	7,40E-04	Mariner-6 transposase; overlaps with monocarboxylate transporter	
**AN11326**	-3,77	2,91E-02	hypothetical protein	
**AN7811**	-3,74	2,96E-02	stcO; sterigmatocystin biosynthesis gene	
**AN1587**	-3,67	3,09E-02	hypothetical protein	
**AN11293**	-3,64	3,14E-02	hypothetical protein	
**AN5326**	-3,62	3,18E-02	conserved hypothetical protein	
**AN11624**	-3,62	3,19E-02	hypothetical protein	
**AN8435**	-3,57	3,30E-02	hypothetical protein	
**AN11403**	-3,54	7,98E-03	hypothetical protein	
**AN10951**	-3,42	3,73E-03	conserved hypothetical protein	
**AN11255**	-3,40	3,67E-02	hypothetical protein	
**AN6001**	-3,35	3,79E-02	asperthicin-biosynthesis gene	
**AN7772**	-3,26	4,04E-02	conserved hypothetical protein with predicted cyt. P450 domain	
**AN7820**	-3,26	4,04E-02	aflR; sterigmatocystin cluster transcriptional activator	
**AN2543**	-3,24	4,09E-02	conserved hypothetical protein similar to rhamnogalacturan lyase	
**AN2483**	-3,22	4,15E-02	conserved hypothetical protein	
**AN11101**	-3,15	4,34E-02	conserved hypothetical protein	
**AN8980**	-3,12	4,43E-02	alcM; ethanol catabolic gene	
**AN10812**	-3,04	4,71E-02	conserved hypothetical protein	
**AN11210**	-3,01	4,80E-02	conserved hypothetical protein	
**AN2804**	-2,97	4,95E-02	conserved hypothetical protein similar to beta galactosidase	
**AN6160**	-2,87	1,97E-03	conserved hypothetical protein similar to Psf2 3´end mRNA processing protein	
**AN8142**	-2,81	2,60E-03	conserved hypothetical protein similar to UbiA-like prenyltransferase	
**AN10746**	-2,80	1,74E-02	conserved hypothetical protein similar to MOSC-domain containing Fe-S cluster delivering enzymes	
**AN10082**	-2,74	3,05E-02	conserved hypothetical protein with Ser/Thr kinase domain	
**AN6736**	-2,72	1,94E-02	conserved hypothetical protein	

1 Gene number according to Fungal Genome Database at Broad Institute
2 ratio between NH_4-repressed and NO_3-induced cells expressed as log2 value
3 established gene function or predicted function according to CADRE Initiative annotation

Gene number[1]	ratio[2]	P-value	known or putative function[3]	
**AN1649**	-2,66	9,76E-03	conserved hypothetical protein	
**AN3409**	-2,63	2,53E-02	hypothetical protein	
**AN7665**	-2,59	4,25E-02	conserved hypothetical protein similar to exosome complex subunit Rrp46	
**AN8323**	-2,49	4,45E-02	conserved hypothetical protein	
**AN0482**	-2,36	1,40E-02	conserved hypothetical protein with ubiquitin-conjugating enzyme domain	
**AN11449**	-2,34	2,71E-02	hypothetical protein	
**AN7728**	-2,30	1,73E-02	conserved hypothetical protein	
**AN1574**	-2,28	3,36E-02	hypothetical protein	
**AN6693**	-2,25	1,24E-02	hypothetical protein	
**AN11121**	-2,24	1,85E-02	hypothetical protein	
**AN0383**	-2,19	2,23E-02	conserved hypothetical protein similar to endo mannanase	
**AN11480**	-2,18	4,09E-02	hypothetical protein	
**AN3549**	-2,16	1,74E-02	conserved hypothetical protein	
**AN10059**	-2,08	3,22E-04	predicted ZN(II)C6 domain containing transcription factor; NirA binding site in 1kb 5´region	
**AN10344**	-2,02	2,89E-05	conserved hypothetical protein with predicted nucleotide-binding domain	
**AN8405**	-2,02	1,94E-02	conserved hypothetical protein with similarity to aspyridone monooxygenase	

Supporting Table 6. *A. nidulans* genes that negatively respond to N-limitation in the wild type

Gene number[1]	ratio[2]	P-value	known or putative function[3]
**AN3396**	-3,69	1,22E-02	conserved hypothetical protein with similarity to panthotenyltrasnferase (NRPS)
**AN1825**	-2,56	2,41E-02	conserved hypothetical protein with similarity to sulfide:quinone oxidoreductase
**AN5734**	-2,45	2,61E-02	conserved hypothetical protein with similarity to MFS-type quinate transporter
**AN0944**	-2,27	3,01E-02	predicted ATP-dependent RNA helicase similar to Rok1
**AN9140**	-2,17	3,27E-02	conserved hypothetical protein with similarity to MFS-type amino acid transporter
**AN1303**	-2,07	2,35E-02	predicted Zn(II)2Cys6-domain containing transcription factor

1 Gene number according to Fungal Genome Database at Broad Institute
2 ratio between NH4-repressed and N-limited cells (60 min.) expressed as log2 value
3 established gene function or predicted function according to CADRE Initiative annotation

Supporting Table 7.: *A.nidulans* strain list.

Strain	Genotype	Reference
**Wild type bio**	*veA1, biA1, yA2*	this study
**Wild type paba**	*veA1, pabaA1*	FGSC
**WT *hER* II/4**	*veA1, argB2* $_{Bgl\ II}$ *, riboA1*	(Pachlinger *et al.*, 2005)
**SAA.111**	*veA1, biA1, Δ argB :: trpC, riboB2, pyroA4, wA3*	(Monahan *et al.*, 2002)
niaDΔ	*veA1, biA1, pyrG89, niaDΔ, wA3*	this study
fhbAΔ	*veA1, biA1, yA2*	this study
fhbBΔ	*veA1, biA1, yA2*	this study
fhbAΔ fhbBΔ	*veA1, biA1,argB2, fhbAΔ::argB, fhbBΔ::argB, yA2*	this study
fhbAΔ fhbBΔ niaDΔ	*veA1, biA1, fhbAΔ::argB, fhbBΔ::argB, niaDΔ*	this study
nirA $^-$	*veA1, pabaA1, nirA637*	(Muro-Pastor *et al.*, 1999)
areA $^-$	*veA1, biA1, pantoB100*	(Kudla *et al.*, 1990)
crnA $^-$ ***crnB*** $^-$	*veA1, biA1, pabaA1,argB2, crnA747, crnB110, argB complemented*	(Unkles *et al.*, 2001) and this study
crnA $^-$ ***crnB*** $^-$ ***niaDΔ***	*veA1, biA1, pabaA1,argB2, crnA747, crnB110, niaDΔ::argB*	this study
niiA $^-$	*veA1, biA1, niiA4, pyroA4, nkuAΔ::bar*	(Cove and Pateman, 1963)
nirAΔ	*veA1, pyrG89, argB2, riboB2, pyroA4, nkuΔ::argB, nirAΔ::riboB fum,*	this study

References for Supporting Table 7

Cove, D.J., and Pateman, J.A. (1963) Independently segregating genetic loci concerned with nitrate reductase activity in *Aspergillus nidulans*. *Nature* **198**: 262-263.

Kudla, B., Caddick, M.X., Langdon, T., Martinez-Rossi, N.M., Bennett, C.F., Sibley, S., Davies, R.W., and Arst, H.N., Jr. (1990) The regulatory gene *areA* mediating nitrogen metabolite repression in *Aspergillus nidulans*. Mutations affecting specificity of gene activation alter a loop residue of a putative zinc finger. *EMBO J* **9**: 1355-1364.

Monahan, B.J., Unkles, S.E., Tsing, I.T., Kinghorn, J.R., Hynes, M.J., and Davis, M.A. (2002) Mutation and functional analysis of the *Aspergillus nidulans* ammonium permease MeaA and evidence for interaction with itself and MepA. *Fungal Genet Biol* **36**: 35-46.

Muro-Pastor, M.I., Gonzalez, R., Strauss, J., Narendja, F., and Scazzocchio, C. (1999) The GATA factor AreA is essential for chromatin remodelling in a eukaryotic bidirectional promoter [published erratum appears in *EMBO J* 1999 May 4;18(9):2670]. *EMBO J* **18**: 1584-1597.

Pachlinger, R., Mitterbauer, R., Adam, G., and Strauss, J. (2005) Metabolically independent and accurately adjustable *Aspergillus sp.* expression system. *Appl Environ Microbiol* **71**: 672-678.

Unkles, S.E., Zhou, D., Siddiqi, M.Y., Kinghorn, J.R., and Glass, A.D. (2001) Apparent genetic redundancy facilitates ecological plasticity for nitrate transport. *EMBO J* **20**: 6246-6255.

Supporting Table 8.: Primers used for cloning and Northern probes.

**primers for *fhbA* and *fhbB* deletion cassette construction**	
**flav 35 Pr F**	GGAAATTGACATCAGCAGTGGGT
**flav 35 Pr R ClaI**	TATATCGATGTGCAAAAAACCGTATTGTCTCA
**flav 35 T F BamHI**	CGAGGATCCCATAACCAGCAGCATTCACGA
**flav 35 T R**	ATCACTCCGGCGAAATCTCATC
**flav 71 Pr F**	ACCACGGTCGCGATTATGAG
**flav 71 Pr R EcoRI**	GAAGAATTCATGATGATGGCTGTCTGTCTGTG
**flav 71 T F ClaI**	TAGATCGATCCATCACGGGAGCTTCAAAACT
**flav 71 T R**	AGCAAAGAAACTCCGAAAAACACC
**arg flavo ko R**	CATGAACTTCCAGCCCTCCTTAG
**arg flavo ko F**	GGGCTATCAAGTCGGGGTCAAT
**arg flavo ko ClaI**	TCGATCGATAGCCATTGCGAAACCTCAGAAG
**flav 35 P F check**	CCGCTGCTCCTTGTCTCTTGA
**flav35 T R check**	TTCGTGAATGCTGCTGGTTA
**flav 71 P F check**	CCCGACATTGAAAGCAGGACAG
**flav 71 T R check**	CGAAAAAAATACAGTCAAATCAGAAGAA
**primers for fhbA and fhbB knock out screening**	
**flav 35 ORF F**	GCCTGCCGCTCTTGCTCAT
**flav 35 ORF R**	CATGATATTGGCTAGCGTCTCCTT
**flav 71 ORF F**	CGAACAAATCCAGCTCATCAAGG
**flav 71 ORF R**	ACGCGACCCTTGTATGTGTAGTTC
**primers for *nirA*Δ deletion cassette construction**	
**nirA Pr-1714_F**	CAACGGATGGTGCTAAGGAAAGAAG
**nirA Pr-1_ClaI_R**	AATCAATCGATGGTAAATCAAGCCCAGACAGATATGTTC
**nirA T+1_BamHI_F**	AATCAGGATCCCAGGCCTATTCACCGCTGCGGATA
**nirA_T+1658_R**	CGGAGAAGAAGCGCATAAGTGATAGG
**primers for *nirA* knock out screening**	
**nirA Pr -1790_F**	GTCTCACGGTCGGGAAGAACAGC
**nirA 557 R**	ATGTATTACCGCATTTGACA
**primers for *niaD*Δ deletion cassette construction**	
**niaD_ATG_F**	ATGTCTACAACCGTCACACAAGTGC
**niaD_1428_EcoRI_R**	AATCAGAATTCGGTATTTGTCTTCGGCGTATTCG
**niaD_1557_BamHI_F**	AATCAGGATCCCGCGCAATGGACGAAGCAC
**niaD_2957_R**	CTCCTCTTTCCATCCCAACGAC
**primers for *niaD* knock out screening**	
**niaD_F**	GCAGCGGGCGGCTGCCAACTG
**arg flavo ko R**	CATGAACTTCCAGCCCTCCTTAG
**niaD_ATG_F**	ATGTCTACAACCGTCACACAAGTGC
**niaD_2957_R**	CTCCTCTTTCCATCCCAACGAC
**primers for Northern probes**	
***niaD* (~ 2950 bp)**	
**niaD ATG F**	ATGTCTACAACCGTCACACAAGTGC
**niaD 2957 R**	CTCCTCTTTCCATCCCAACGAC
***niiA (~* 1320 bp)**	
**niiA F**	CATCATGCCGTTGCTGGACG
**niiA R**	GCTTAGGTCCGGCCTGTTG
***fhbA* (~ 1040 bp)**	
**flav 71 ORF F**	CGAACAAATCCAGCTCATCAAGG
**flav 71 ORF R**	ACGCGACCCTTGTATGTGTAGTTC
***acnA* (~ 750 bp)**	
**actin F**	GATCGGTATGGGTCAGAAGGA
**actin R**	CGATGTTGCCGTACAGATCC
***18S* (1600 bp)**	
**18S F**	GAATGGCTCATTAAATCAGTTATCG
**18S R**	CGGGTTTAACCAGCTTTCCGGC
